# Whole-genome sequence association analysis of blood proteins in a longitudinal wellness cohort

**DOI:** 10.1186/s13073-020-00755-0

**Published:** 2020-06-23

**Authors:** Wen Zhong, Anders Gummesson, Abdellah Tebani, Max J. Karlsson, Mun-Gwan Hong, Jochen M. Schwenk, Fredrik Edfors, Göran Bergström, Linn Fagerberg, Mathias Uhlén

**Affiliations:** 1 Science for Life Laboratory, Department of Protein Science, KTH-Royal Institute of Technology, Stockholm, Sweden; 2 Department of Molecular and Clinical Medicine, Institute of Medicine, Sahlgrenska Academy, Gothenburg University, Gothenburg, Sweden; 3 Department of Clinical Genetics and Genomics, Sahlgrenska University Hospital, Gothenburg, Sweden; 4 Department of Clinical Physiology, Sahlgrenska University Hospital, Gothenburg, Sweden; 5 Department of Neuroscience, Karolinska Institutet, Stockholm, Sweden

**Keywords:** Protein levels, Blood, Genetics, Whole-genome sequence, Genome-wide associations

## Abstract

**Background:**

The human plasma proteome is important for many biological processes and targets for diagnostics and therapy. It is therefore of great interest to understand the interplay of genetic and environmental factors to determine the specific protein levels in individuals and to gain a deeper insight of the importance of genetic architecture related to the individual variability of plasma levels of proteins during adult life.

**Methods:**

We have combined whole-genome sequencing, multiplex plasma protein profiling, and extensive clinical phenotyping in a longitudinal 2-year wellness study of 101 healthy individuals with repeated sampling. Analyses of genetic and non-genetic associations related to the variability of blood levels of proteins in these individuals were performed.

**Results:**

The analyses showed that each individual has a unique protein profile, and we report on the intra-individual as well as inter-individual variation for 794 plasma proteins. A genome-wide association study (GWAS) using 7.3 million genetic variants identified by whole-genome sequencing revealed 144 independent variants across 107 proteins that showed strong association (*P* < 6 × 10^−11^) between genetics and the inter-individual variability on protein levels. Many proteins not reported before were identified (67 out of 107) with individual plasma level affected by genetics. Our longitudinal analysis further demonstrates that these levels are stable during the 2-year study period. The variability of protein profiles as a consequence of environmental factors was also analyzed with focus on the effects of weight loss and infections.

**Conclusions:**

We show that the adult blood levels of many proteins are determined at birth by genetics, which is important for efforts aimed to understand the relationship between plasma proteome profiles and human biology and disease.

## Background

The levels of blood proteins are important as a measure of human health and disease, and protein assays are therefore used frequently in diagnostics. In the future, such assays hold great promise for precision medicine efforts to detect early signs of disease and to stratify and monitor patients. An important issue linked to blood analysis is the underlying effect of genetics to determine stable differences in protein levels between individuals. The levels of blood proteins have previously been determined to be influenced both by genetic and environmental factors, as studied by mass spectrometry-based proteomics [1–4], nucleic-acid based assays [5–8], and immuno-based assays [9–14]. Effects based on sex [15], specific diets [15], age [16], and infections [17] have also been reported suggesting an important role for quantitative blood protein assays for individualized diagnosis of health and disease.

Romanov et al. [15] showed that based on mass spectrometry analysis and genetic variability analysis, the genetic and environmental effects of proteotypes of individuals could be disentangled. At most 13.5% of the observed differences of protein levels could in this study be explained by sex, genetics, and diet. Similarly, Wu et al. [2] showed the genetic component of protein levels using tag-based quantitative mass spectrometry from lymphoblastic cell lines from individuals genotyped in the HapMap project by identification of cis-acting protein quantitative trait loci (pQTLs). Furthermore, the quantitative variability of 324 plasma proteins were analyzed by Liu et al. [3] in a human twin population and showed different patterns of abundance variability with genetics effecting the protein levels. Solomon et al. [4] identified 109 independent associations (36 protein and 73 peptide) using whole-exome sequencing and mass spectrometry in 165 participants of the Tromsø study. Their data suggested that the plasma concentration of clinical biomarkers needs to be calibrated against genetic and temporal factors. These studies show that genetics is an important factor for understanding individual variation of protein concentration levels in human blood.

To complement these studies based on mass spectrometry analysis, several genome association studies have recently been conducted involving multiplex protein analysis using aptamer/SOMAmer reagents analysis [5–8] or protein extension analysis (PEA) [9–12]. Sun et al. [5] applied an integrative approach to link genetic variation determined by an Affymetrix microarray platform with protein levels determined with a SOMAmer platform to determine genetic factors to diseases via protein levels, highlighting opportunities to match existing drugs with disease indications. Similarly, Emilsson et al. [6] measured the protein levels of individuals over 65 years of age using genotyping and a SOMAmer platform and identified many pQTLs associated with complex diseases. Carayol et al. [7] identified 55 BMI-associated pQTLs using SOMAscan proteomic assay and genotyping in 494 obese subjects. Suhre et al. [8] analyzed the associations between protein levels and gene variants in a German cohort using SOMAscan platform and Affymetrix Array and identified 57 genetic risk loci for 42 disease end points. The PEA platform has also been used for genetic association studies, such as the identification of 16 pQTLs associated with known biomarkers [9], 79 loci for plasma protein biomarkers in cardiovascular disease [10], 8 cis-pQTL in the InCHIANTI study [11], 41 loci for the plasma levels of neurological proteins [12], and 131 independent sequence variant associations of the cardiometabolic proteome [13]. In addition, Yao et al. [14] analyzed the association of protein levels and genetic factors for 16,000 pQTL variants in more than 6000 individuals in the Framingham Heart Study using Luminex multiplex immunoassays and identified 13 proteins harboring pQTL variants that match coronary disease-risk variants from GWAS.

Here, we have for the first time in a longitudinal study combined whole-genome sequencing with multiplex protein analysis to investigate the effect on genetic variability on protein levels in blood. A cohort of 101 healthy individuals between 50 and 65 years of age have been studied longitudinally for 2 years with repeated analysis to identify genetic associations with consequences for protein blood levels. A large number of anthropometric and clinical variables based on classic clinical chemistry analysis have also been assessed to probe the importance of environmental factors to protein variability. The study has identified a set of proteins in which the difference in concentration levels between individuals is heavily influenced by the genetic architecture of the individual. Most of these differences are stable during the study period, suggesting that genetic factors are important to define the levels of these proteins in blood throughout adult life.

## Methods

### The Swedish SciLifeLab SCAPIS Wellness Profiling (S3WP) study

The S3WP study is based on the Swedish CArdioPulmonary bioImage Study (SCAPIS) with 30,154 individuals enrolled at ages between 50 and 64 years recruited from random sampling of the general Swedish population [18, 19]. A total of 101 healthy individuals were recruited in the S3WP study and followed longitudinally for 2 years with repeated analyses of molecular markers in blood and stool samples in combination with physical measurements. Examinations in SCAPIS include imaging to assess coronary and carotid atherosclerosis, clinical chemistry, anthropometry, and extensive questionnaires, as previously described [18]. Thus, the subjects had been extensively phenotyped before entering the S3WP study. In SCAPIS, no exclusion criteria are applied except the inability to understand written and spoken Swedish for informed consent. In the S3WP study, exclusion criteria include (1) previously received health care for myocardial infarction, stroke, peripheral artery disease, or diabetes; (2) presence of any clinically significant disease which, in the opinion of the investigator, may interfere with the results or the subject’s ability to participate in the study; (3) any major surgical procedure or trauma within 4 weeks of the first study visit; or (4) medication for hypertension or hyperlipidemia. The study is approved by the Ethical Review Board of Göteborg, Sweden. All participants provided written informed consent. The study protocol conforms to the ethical guidelines of the 1975 Declaration of Helsinki.

### Study design and sample collection

Subjects in the S3WP study were examined and sampled every third month (± 2 weeks) in the first year and approximately a 6-month interval in the second year. All subjects were fasting overnight (at least 8 h) before the visits. Identical examinations were performed at each visit, including anthropometric measurements, body fat using bioimpedance and blood pressure. A selection of questions from the initial SCAPIS questionnaire was repeated to note any changes in health and lifestyle factors between each visit. Each visit also included collection of blood, urine, and feces for subsequent clinical chemistry and omics analyses. All samples were stored at − 80 °C until use. From visit 2 and onward, subjects were wearing accelerometers (Polar A360) to measure physical activity.

### Anthropometric measurements

Height was measured in indoor clothing to the nearest centimeter without shoes. Weight was measured on a calibrated digital scale, with subjects dressed in light indoor clothing without shoes. The body mass index (BMI) was calculated by dividing the weight (kg) by the square of the height (m). Waist circumference was measured midway between the palpated iliac crest and the palpated lowest rib margin in the left and right mid-axillary lines. Hip circumference was measured at the maximum circumference over the buttocks. Bioimpedance was measured using Tanita MC-780MA according to the manufacturer’s instructions. Systolic and diastolic pressure was registered in supine position and after 5 min of rest, using the automatic Omron P10. The blood pressure was measured in both arms at visit 1 and thereafter in the arm that showed the highest blood pressure at visit 1.

### Clinical chemistry

Clinical chemistry and hematology measurements included plasma glucose, hemoglobin A1c (HbA1c), triglycerides (TG), total cholesterol, low-density lipoprotein (LDL), high-density lipoprotein (HDL), apolipoprotein A1 (ApoA1), apolipoprotein B (ApoB), ApoA1/B ratio, creatinine, high sensitive C-reactive protein (hsCRP), alanine aminotransferase (ALAT), gamma-glutamyltransferase (GGT), urate, cystatin C, vitamin D, troponin T (TNT), N-terminal pro-brain natriuretic peptide (NT-proBNP), hemoglobin (Hb), and a complete blood count with differential. In total, a variety of 30 clinical chemistry parameters were included in the study; see for more details Additional file 1: Table S1.

### Whole-genome sequencing

Genomic DNA was quantified using Qubit 2.0 Fluorometer (Invitrogen), fragmented into average 350-bp fragments using E220 focused-ultrasound sonicator (Covaris), and 1 μg of fragmented DNA was converted into sequencing ready library using TruSeq DNA PCR-free HT Sample preparation method (Illumina). The obtained library was quantified using KAPA SYBR FAST qPCR (Kapa Biosystems) and pair-end (2 × 150 bp) sequenced to average 30× coverage on the HiSeq X system (Illumina) using v2 flowcells. Demultiplexing was done without allowing any mismatches in the index sequences. Bioinformatic analysis of the sequence data was carried out using Mutation Identification Pipeline (version 4.0.18) [20]. Briefly, alignment was done using BWAmem using reference genome GRCh38.p7, and single-nucleotide and insertion/deletion variants called using GATK best practices pipeline (https://software.broadinstitute.org/gatk/best-practices, GATK v3.6). Structural variants were called using Manta (v1.0.3) [21]. Variants in the any of the 56 ACMG genes [22] were excluded from further analysis in order to avoid secondary findings.

The VCF files were then converted to PLINK-format with the PLINK software, version 19 [23]. Quality control (QC) was conducted to avoid false findings. The exclusion criteria for variants include (1) remove individuals with high missing genotype rates (> 5%), (2) remove SNPs fail the genotyping rate threshold 0.05, (3) remove SNPs with low minor allele frequencies (MAF) (< 5%), and (4) remove SNPs fail the Hardy-Weinberg equilibrium (HWE) test (*P* < 0.001). In total, 7,275,131 high-quality variants were identified in all samples from 101 individuals with a general genotyping rate of 99.93%. The multidimensional scaling (MDS) analyses of the pairwise identity-by-state (IBS) distance of the samples was conducted within PLINK.

### Plasma protein profiling

We used a multiplex proximity extension assay (Olink Bioscience, Uppsala Sweden) [24] to measure the relative concentrations of plasma proteins in the study. Each kit provides a microtiter plate for measuring 92 protein biomarkers in all prepared samples. Each well contains 96 pairs of DNA-labeled antibody probes. Samples were incubated in the presence of proximity antibody pairs tagged as previously described. To minimize inter- and intra-run variation, the samples were randomized across plates and normalized using both an internal control (extension control) and an inter-plate control and then transformed using a pre-determined correction factor. The pre-processed data were provided in the arbitrary unit Normalized Protein eXpression (NPX) on a log2 scale, and a high NPX presents high protein concentration. In this study, eleven Olink panels have been used including Cardiometabolic, Cell Regulation, Cardiovascular II (CVD II), Cardiovascular III (CVD III), Development, Immune Response, Oncology II, Inflammation, Metabolism, Neurology, and Organ Damage. Quality control (QC) was performed at both sample and protein levels. A sample will flag (not pass the QC) if incubation control deviates more than a pre-determined value (± 0.3) from the median value of all samples on the plate (www.olink.com). To reduce the batch effect between samples run at different times, bridging reference samples from different visits were also run on plates from the different batches. Reference sample normalization based on bridging samples was conducted to minimize technical variation between batches (www.olink.com).

Two strategies were used to assess the batch effect: (1) the ratio of maximum and minimum interquartile range (IQR) of protein concentrations across six visits and (2) three-way analysis of variance (ANOVA) analysis of protein concentrations for factor batch number, factor visit, and factor subject. Proteins with the ratio of maximum and minimum IQR > 1.8 or coefficient of sampling date from ANOVA > 10 were considered to have a problematic batch effect and were removed from the dataset. Thirty-nine replicated proteins from multiple panels were also removed. The filtering process resulted in a total of 794 unique proteins for 90 subjects and 6 visits (540 samples) in the analysis of the study (Additional file 1: Table S2).

### Genome-wide association analysis

Baseline protein concentration level for each subject was calculated as a median of NPX values across 6 visits. No significant association between protein levels and ancestry was observed by using mixed effect modeling in the study. Therefore, no correction for ancestry was applied. Association between each protein and genetic variant was performed using a linear regression model adjusted for age and gender at baseline using PLINK v1.9 [23]. Bonferroni-adjusted *P* value < 6 × 10^−11^ (genome-wide threshold of *P* = 5 × 10^−8^, 798 proteins tested) were considered to be significant in the study. Functional annotation of variants was performed using Ensembl Variant Effect Predictor (VEP) v87 [25]. A cis-pQTL variant was defined as a SNP residing within 1 megabase (Mb) upstream or downstream of the transcription start site of the corresponding protein-coding gene. A SNP located > 1 Mb upstream or downstream of the gene transcript or on a different chromosome from its associated gene was categorized as a trans-pQTL variant. Linkage disequilibrium (LD) was computed as the square of Pearson’s correlation (*r*^2^) between genotype allele counts across 101 subjects. To identify independent pQTLs for a given protein, LD *r*^2^ > 0.1 with window size 1 Mb was first used to exclude the correlated variants. For proteins with multiple pQTLs, a conditional analysis was then carried out in which the genetic associations were re-calculated using the sentinel SNP as covariate. Only associations with conditional *P* value < 0.01 were considered to be independent pQTLs.

### Replication of previous pQTLs associated with blood proteins

Experimental Factor Ontology (EFO) term “blood protein measurement” (EFO_0007937) was used for the search in NHGRI-EBI GWAS Catalog (accessed February 2020) with the exclusion of child trait datasets and non-European studies. A total of six studies were identified, including Yao et al. [14], Melzer et al. [11], Hillary et al. [12], Suhre et al. [8], Emilsson et al. [6]**,** and Sun et al. [5]. In addition, by using literature search for pQTL studies, Enroth et al. [9], Folkersen et al. [10], Liu et al. [3], and Johansson et al. [1] were also included in the analysis. In total, 3751 pQTLs from 10 studies were included in the analysis. The replication of pQTL was considered if SNP had a correlation of *r*^2^ > 0.6 and associated with the same protein in our study (Additional file 2: Table S4). Replication *P* values were calculated using weighted meta-analysis implemented in METAL [26].

### Overlap of cis-pQTL with cis-eQTL

Each independent cis-pQTL variant was queried against publicly available eQTL association data using PhenoScanner [27]. Non-European studies and non-blood tissues were excluded manually. For each eQTL, only the entry with strongest association among the pQTL variants was present (Additional file 2: Table S5).

### Disease associations

We examined whether the sentinel variants or their strong proxies (LD *r*^2^ > 0.8) were associated with human diseases using PhenoScanner [27] with default parameters. Non-European studies and non-disease phenotypes such as anthropomorphic, molecular, and physiological traits were excluded. For each disease, only the entry with strongest association among the pQTL sentinel variants or their proxies were reported (Additional file 2: Table S6).

### Hierarchical clustering and canonical correspondence analysis

The hierarchical clustering results visualized in dendrograms are based on Pearson correlation and were created by first calculating a correlation matrix of Pearson’s ρ between all 540 samples. The correlation was converted to a distance metric (1 – ρ) and was clustered using unsupervised top-down hierarchical clustering, where at each stage the distances between clusters are recomputed by the Lance-Williams dissimilarity update formula according to average linkage. Canonical correspondence analysis (CCA) was performed on the NPX values for all 794 proteins in 540 samples with clinical chemistry/anthropometric measurements as constraining variables using the “vegan” package in R v3.5.3 [28]. CCA functions are based on Legendre & Legendre’s algorithm [29]: in CCA, chi-square transformed data matrix is subjected to weighted linear regression on constraining variables, and the fitted values are submitted to correspondence analysis performed via singular value decomposition (SVD).

### Statistical analysis

Mixed-effect modeling was performed using the lme4 package [30]**,** and Kenward-Roger approximation [31] was used to calculate *p* values which were subsequently adjusted for multiple testing using Benjamini-Hochberg method [32]. *p* values were considered significant if less than 0.01. Variance analysis of the protein levels was conducted using multiple linear regression model with all protein significantly associated pQTLs, clinical chemistry/anthropometric parameters, sex, and visit as variables in the model. The fraction of explained variability was measured as the Sum of Squares Explained (SSE) and was determined using ANOVA. All of the data analysis was performed using the R project [33].

## Results

### The study cohort and clinical chemistry

**Fig. 1:**
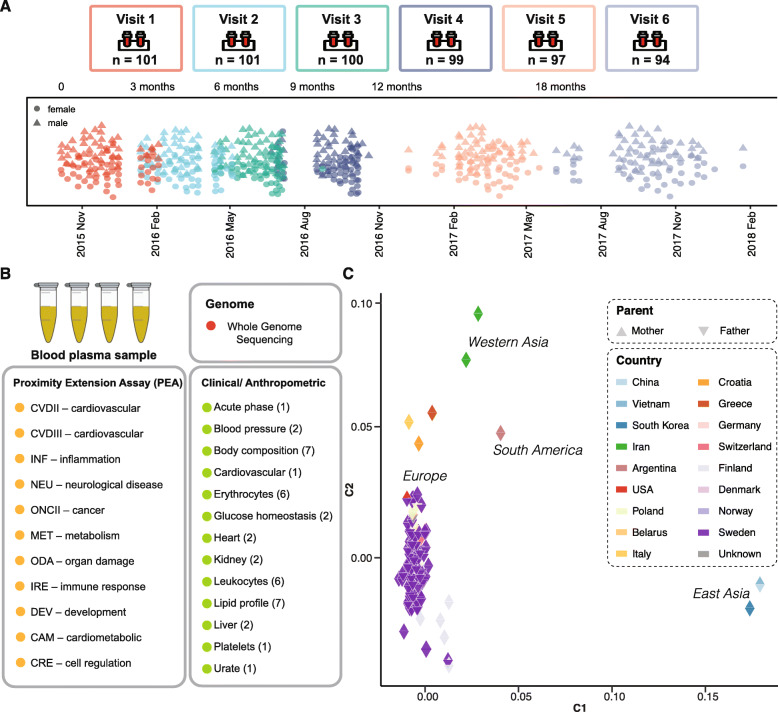
Overview of the study. **a** In total, 101 subjects were included in the study. The upper part shows the number of individuals that came to each of the six visits (red, blue, green, purple, orange, and gray). The lower part shows the distribution of each visit for the subjects that completed the program across 2 years. **b** The rectangular plot shows the types of data that is collected in the study; see more details in Table S2. **c** The MDS plot shows the pairwise genetic distances between 101 subjects based on the whole genome sequencing. The color code indicates the origin of the parents of each individual (upwards triangle, mother; inverted triangle, father)

A total of 101 individuals were recruited from the SCAPIS study [18], including 48 males and 53 females between 50 and 65 years of age (Fig. 1a). Among them, 92 (91%) individuals were of European descent, while a few were of South American or Asian origin. Extensive phenotype characterization of the subjects was conducted before the study to establish the inclusion and exclusion criteria for the definition of “healthy” subjects. The sample collection in combination with clinical chemistry analysis of 30 parameters and as well as anthropometric measurements was conducted every 3 months in the first year and at approximately a 6-month interval in the second year (Fig. 1b). The complete list of assessed clinical variables is available in Additional file 1: Table S1. Among the 101 subjects, 94 completed the full 2-year study including six visits.

### Whole-genome sequencing

DNA from whole blood of each individual was isolated at the first visit and the whole genome was determined using next-generation sequencing. All 101 individuals passed the quality control. In total, 7.3 million variants were identified with a general genotyping rate of 99.93%. A MDS analysis was performed based on the genome-wide IBS pairwise distances of the total set of variants from the 101 individuals (Fig. 1c). Distinct subsets of individuals revealed the relationship of geographic origin of the parents.

### Plasma protein profiling

**Fig. 2:**
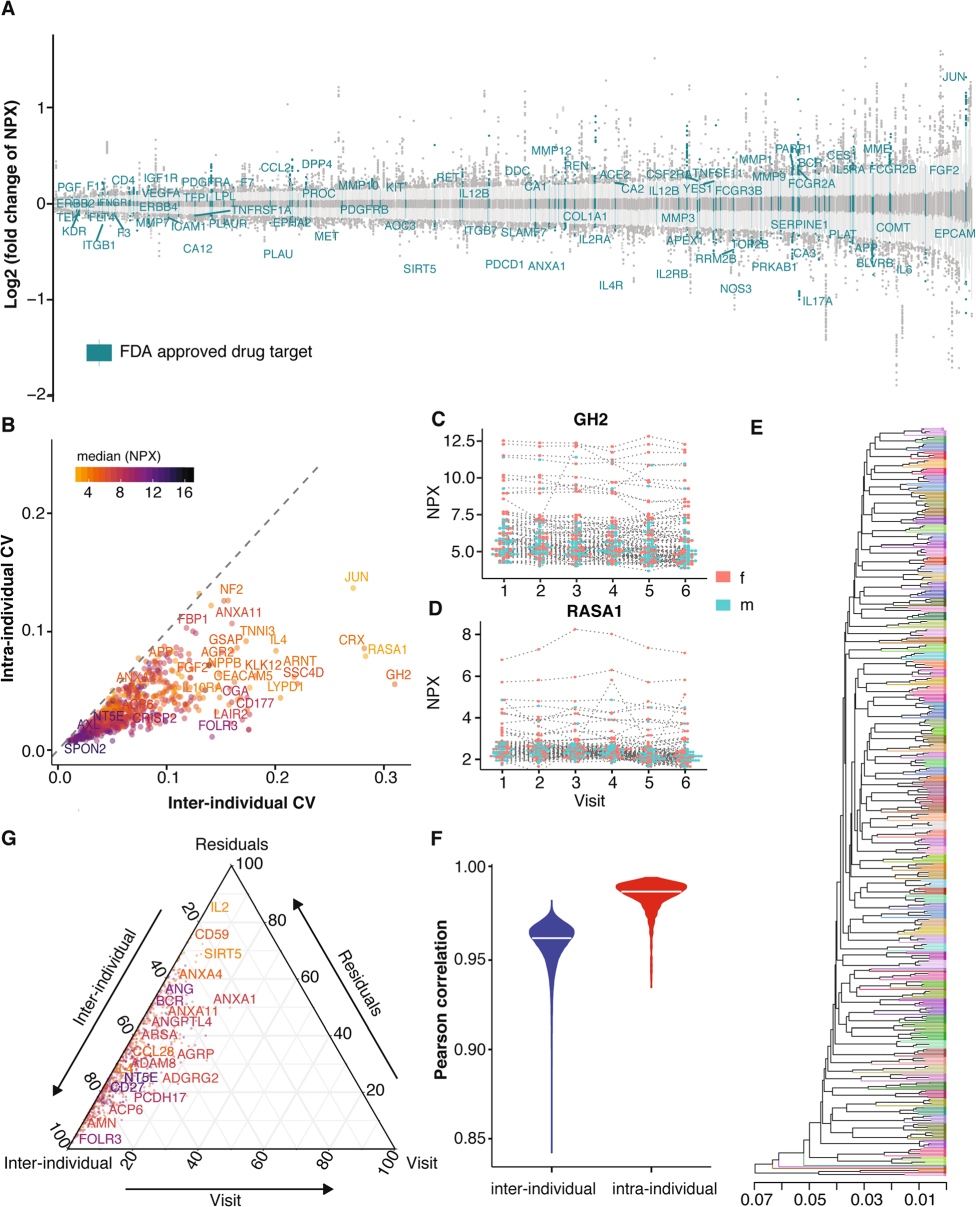
Longitudinal plasma protein profiling. **a** The distribution of the Log_2_ fold change of protein concentration per sample versus the average protein concentration level with FDA approved drug targets highlighted. **b** The inter-individual and intra-individual variation of protein levels calculated as the coefficient of variation (CV) for each protein within each visit and across all analyzed individuals (*n* = 90), and as the mean CV for each protein within each individual across all visits (*n* = 6), respectively, colored by the median concentration level of protein. The protein concentration variation across visits one to six, with each individual connected with a dotted line for **c** growth hormone 2 (GH2) and **d** RAS p21 protein activator 1 (RASA1). The color code indicates females and males. **e** Hierarchical clustering based on pairwise Pearson correlation distance of the protein concentration in all 540 samples is shown with labels color coded by individual (see more details in Fig.S3). **f** Violin plot showing the distribution of inter- and intra-individual Pearson correlation for all samples. **g** Ternary plot based on two-factor ANOVA for all proteins, assessing the relative effect of the inter-individual variation, visits, and residuals. The color code indicates the median concentration level of protein

The protein levels of plasma samples from the subjects were analyzed using PEA as described previously [24]. All samples were analyzed with eleven panels as outlined in Fig. 1b covering plasma proteins of interest for cardiovascular and neurological disease, inflammation, cancer, metabolism, organ damage, development, and cell regulation. Bridging reference samples were used for inter-plate normalization (Additional file 1: Fig. S1A), and the comparison of reference samples run on different plates showed a strong correlation among different replicates (Additional file 1: Fig. S1B). Reference sample normalization was conducted to reduce the batch effect (Additional file 1: Fig. S1C-D, see more details in the “Methods” section). Proteins run in multiple panels were also analyzed and found to correlate well with an average Pearson correlation between panels of 0.86 (Additional file 1: Fig. S2A), as exemplified by the interleukin-6 protein which was run in four different panels (Additional file 1: Fig. S2B). In total, the relative protein concentration levels of 794 unique protein targets for 90 subjects with six visits were generated. Among them, 80 proteins are found in the list of drug targets for FDA approved drugs [34] (Fig. 2a, Additional file 1: Table S2).

To assess the variability of protein concentration, we compared the IQR of the fold change of protein concentration levels from their median abundance level (Fig. 2a, Additional file 1: Table S2). The most variable protein in the study was kallikrein-related peptidase 12 (KLK12) which is involved in angiogenesis. Spondin 2 (SPON2), a cell adhesion protein that promotes adhesion and outgrowth of hippocampal embryonic neurons, on the other hand, was the most stable protein with a median fold change of 1 and IQR of 0.01. Extreme outliers were also observed, suggesting the discrepancy in protein concentration levels among individuals. The inter-individual variation (calculated as average coefficient of variation (CV)) and the intra-individual variation of each protein for each individual across the six visits were also determined (Table S2). Figure 2b shows that the majority of all proteins have higher variation between individuals rather than within individuals. Growth hormone 2 (GH2) and RAS p21 protein activator 1 (RASA1) are the most dispersed proteins on inter-individual level. The overview of the concentration levels across six visits for these two proteins is visualized in Fig. 2c and d respectively. The concentration of both proteins was relatively stable across the six visits for each individual, and distinct groups of individuals with elevated concentration levels can be identified based on the longitudinal protein concentration profiles.

### Clustering analysis of the protein profiles

Unsupervised clustering analysis was performed based on the Pearson correlation of the global protein concentration profiles based on six samples for each of the 90 individuals. The hierarchical tree shows that the majority of samples from the same individual cluster together, indicating that the intra-individual variation is smaller than the inter-individual variation in normal healthy individuals (Fig. 2e, Additional file 1: Fig. S3). The comparison of the distribution of intra-individual and inter-individual correlations also demonstrates a similar conclusion with a median intra-individual correlation of 0.99 and median inter-individual correlation of 0.96 (Fig. 2f). The effect of the inter-individual variation, visits, and residuals for each of the 794 proteins was assessed using two-factor ANOVA, and the proportion of variance explanation is visualized as a ternary plot (Fig. 2g). The plot demonstrates that most variability can be observed between individuals (inter-individual) with relatively low contribution by the visits factor. Folate receptor 3 (FOLR3) shows the largest inter-individual differences with 99.4% variance explained by subjects, 0.1% by visits, and 0.5% by residuals.

A small number of individuals (*n* = 10) showed a higher variability between some of the visits, and these can be seen as outliers in the hierarchical tree (Fig. 2e and Additional file 1: Fig. S3), as one or more visits are not clustering with the others from the same individual. Pairwise comparisons of the protein levels across six visits of the 10 individuals were shown in Additional file 1: Fig. S4. Interestingly, one of the individuals (W0010) started a dietary change after visit two and thus lost weight between visit three (120.5 kg) and visit four (104.7 kg) (Additional file 1: Fig. S5A). For another individual (W0022), the clinical chemistry result reveals elevated C-reactive protein (CRP) levels (79 mg/L) at visit two due to an infection (Additional file 1: Fig. S5B). An analysis of the protein profiles of these two individuals will be described more in depth below.

### Genome-wide association analysis of the blood protein profiles

**Fig. 3:**
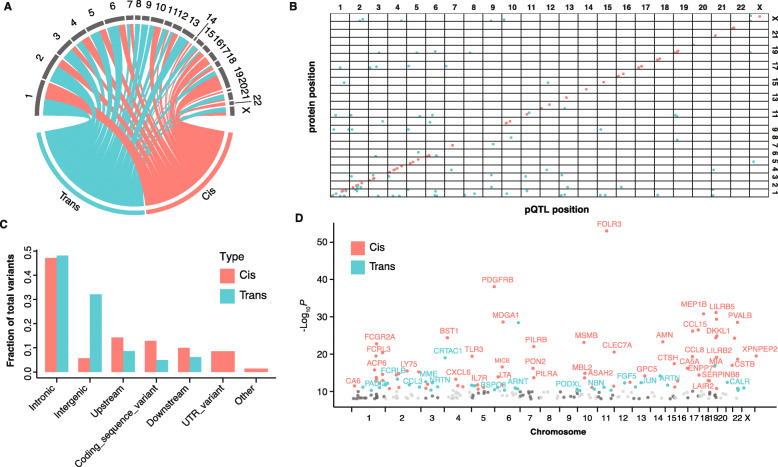
Global analysis of the genetic regulation of the proteome. **a** Chord diagram showing the distributions of cis- and trans-pQTLs in 23 chromosomes. Each link represents cis- or trans-pQTLs in a chromosome, respectively, with the ribbon width reflecting the number of pQTLs. **b** Genomic locations of the pQTL variants and the associated proteins, colored by cis- and trans-pQTLs. **c** The fractions of cis- and trans- pQTLs in different types of genomic regions. **d** Manhattan plot of the sentinel pQTL per protein. The color code indicates the cis- and trans-pQTLs for the 107 proteins with significant associations, and the gray dots represent the none significant associations

To investigate the genetic influences on inter-individual differences in blood protein concentration, a genome-wide association analysis based on 7.3 million variants identified by whole-genome sequencing and 794 plasma protein profiles was performed. A total of 2936 associations reached a given statistical significance level (*P* < 6 × 10^−11^) (Additional file 1: Fig. S6). Among them, 144 significant associations between 107 proteins and 143 independent genetic variants (LD *r*^2^ < 0.1, conditional *P* < 0.01) were identified (Fig. 3a), including 67 cis-pQTL variants for 67 proteins and 77 trans-pQTL variants for 40 proteins (Fig. 3b). Among them, 74% of the pQTLs including the proxy of the pQTLs (LD *r*^2^ > 0.6) have not been reported before. All but 13 of the pQTLs replicated at nominal significance (*P* < 0.001) in previous studies (see more details in methods and Additional file 2: Table S4). Most of the cis-pQTLs and trans-pQTLs were found in intronic, intergenic, or other untranslated regions (Fig. 3c). The association between cis- or trans-pQTL with genomic regions was further examined by using Fisher’s exact test. We found that cis-pQTL variants were higher enriched in coding regions (*P* < 0.1) and untranslated regions (*P* < 0.01), while trans-pQTL variants were higher enriched in intergenic regions (*P* < 0.001). In addition, 45% (*n* = 30) of the cis-pQTLs also had an eQTL for the same protein in blood (Additional file 2: Table S5), suggesting that the genetic effect on plasma protein levels is mainly on transcription level. Sentinel pQTL variant was determined as the variant with lowest *P* value at each pQTL locus and visualized in Fig. 3d. The variants are relatively equally distributed between the chromosomes for both cis- and trans-pQTLs. To investigate the associations between pQTLs and human diseases, we also examined whether the sentinel variants or variants in LD *r*^2^ > 0.8 were identified in disease-GWAS studies. In total, 16 pQTLs were associated with 21 diseases (Additional file 2: Table S6). For example, rs6727306 was identified as an atopic dermatitis risk loci in a multi-ancestry GWAS study [35]. Here, we also show the association of rs6727306 between interleukin 18 receptor 1 (IL18R1) which contributes to IL18-induced cytokine production [36].

**Fig. 4:**
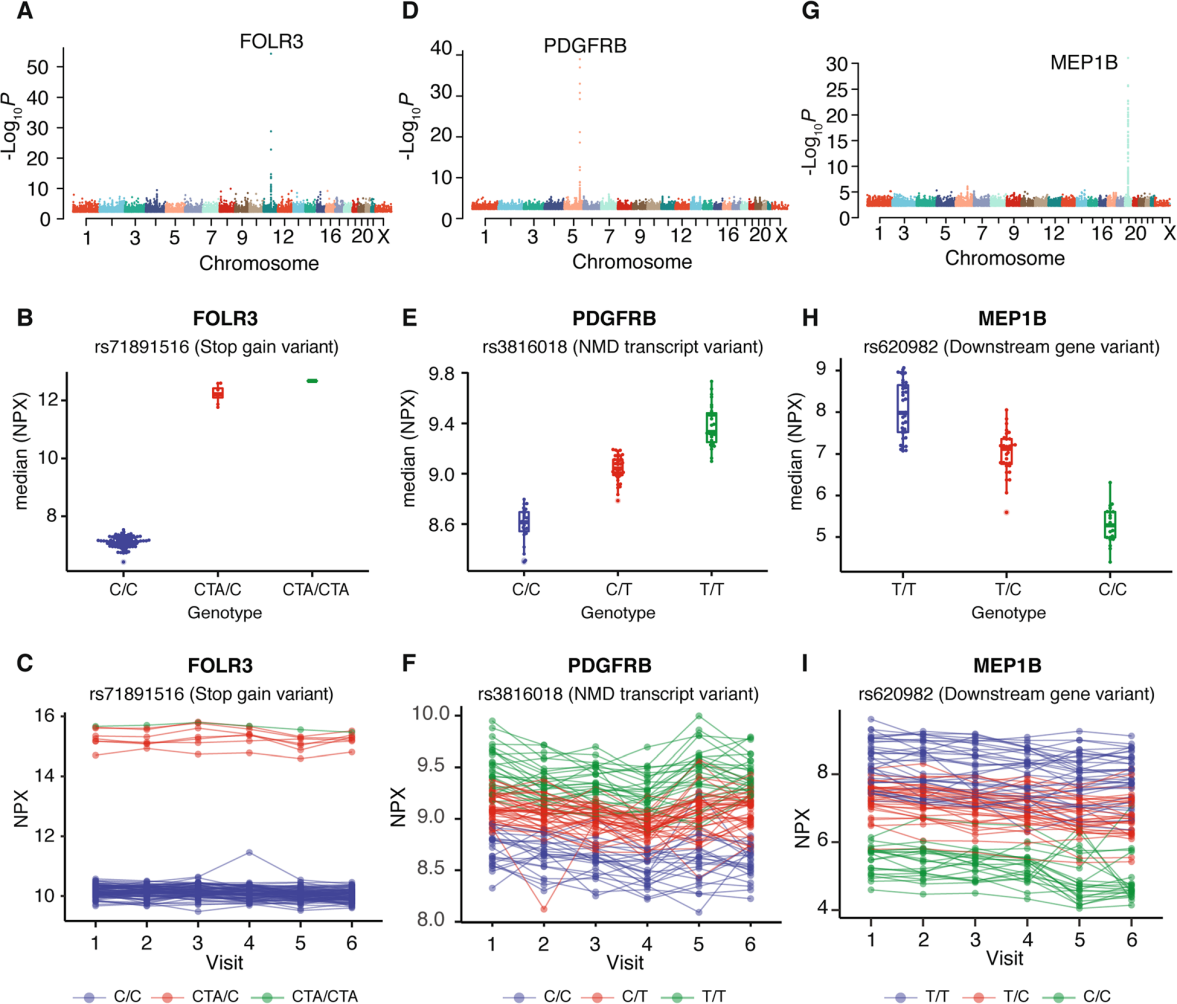
Examples of three proteins with the top most significant pQTLs. **a** Manhattan plot of protein FOLR3 shows the genome locations of all associated pQTLs. **b** Bee-swarm and box plot of protein FOLR3 shows the association between genotype of rs71891516 with median concentration of FOLR3. **c** The longitudinal protein concentration across visits one to six with each individual connected with a dotted line for FOLR3. **d** Manhattan plot for protein PDGFR3. **e** Bee-swarm and box plot showing the associations between genotype of rs3816018 with median concentration of PDGFRB. **f** Longitudinal protein concentration levels of PDGFR3. **g** Manhattan plot for protein MEP1B. **h** Bee-swarm and box plot showing the associations between genotype of rs3816018 with median concentration of MEP1B. **i** Longitudinal protein concentration levels of MEP1B. The color indicates the genotypes of rs71891516, rs3816018 and rs620982, respectively

In Fig. 4, the three proteins with strongest associations between blood protein levels and genetic variants are analyzed in more depth. The genetic variants associated with the concentration levels of the FOLR3 protein (Fig. 4a) are all found at chromosome 11 (cytoband q13.4) in close proximity to the gene coding for FOLR3. The highest association is found for variant rs71891516, which is a stop gain variant in the coding region of FOLR3. FOLR3 is a secreted plasma protein [37] that can bind to folate and reduce folic acid derivatives and mediate delivery of 5-methyltetrahydrofolate to the interior of cells [38]. Interestingly, individuals that carry the variant thus will have a premature termination codon which signals the end of translation. This interruption causes the protein to be abnormally shortened. A more detailed analysis of the two chromosomes of the individuals reveals that the protein levels are high for both the homozygote and heterozygotes for the stop-gain variant (Fig. 4b). The longitudinal analysis during the six visits for the 90 individuals (Fig. 4c) demonstrates that the individual protein levels were remarkably stable during the 2-year period. The reason behind the difference in levels is not known at present, but it is tempting to speculate that the shorter version has longer blood half-life and thus yields higher concentration levels in blood. In this context, it is important to note that the truncated variant of FOLR3 might have an altered antibody binding, and therefore, the apparent change in concentration is instead due to altered epitope binding. This needs to be ruled out by more in-depth analysis using antibody-independent analysis.

For the protein platelet-derived growth factor receptor beta (PDGFRB), the genetic variants (Fig. 4d) are located to chromosome 5 (cytoband q32), which is the location of the protein-coding gene. The highest association is found for variant rs3816018, which has been previously reported in Garrigos et al. [39] and Benson et al. [40]. Interestingly, the chromosomal analysis shows that heterozygote individuals for the protein variant have intermediate levels of blood protein levels (Fig. 4e) compared to the homozygotes. Similarly, to FOLR3, most of the relative levels of the individuals were stable during the 2-year study period (Fig. 4f). For the protein meprin A subunit beta (MEP1B), the genetic variants (Fig. 4g) are located to chromosome 18 (cytoband q12.1), which again is the location of the protein-coding gene. The highest association is found for variant rs620982, located downstream of the MEP1B gene. Again, the heterozygote individuals have intermediate levels of the protein (Fig. 4h), and these levels are stable during the 2-year study period (Fig. 4i).

### Integrative multivariate data analysis

**Fig. 5:**
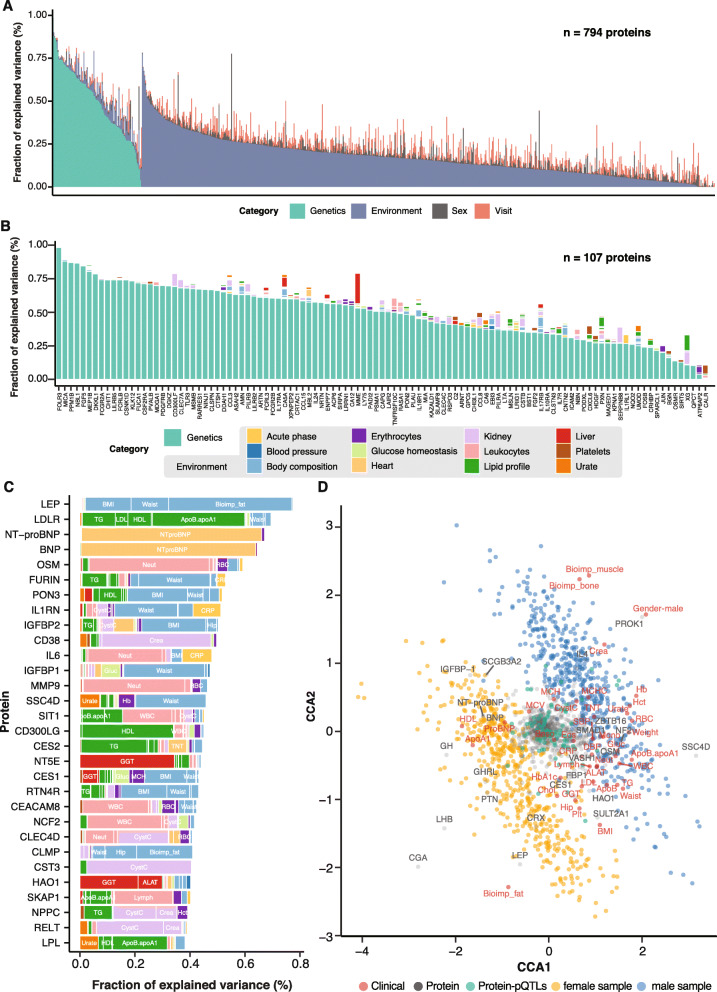
Influence of genetic and environmental factors on the blood protein level variability. **a** Barplot of variance explanation fraction of each component for 794 proteins (green: Genetics; purple: Environmental; gray: Sex; red: Visit) determined by a linear mixed model. **b** Barplot of variance explanation fraction of each component for 107 proteins, color coded by different variables. **b** Barplot of the top 30 proteins most strongly associated with environmental components, with the most significant variables labeled and using the color code in (**b**). **c** Canonical correspondence analysis (CCA) triplot showing correlations between protein levels and the clinical or anthropometric variables, as well as all individual samples

To get a comprehensive quantification of the effects of genetic and non-genetic factors on the variation of protein concentration during the longitudinal study period, we established a linear-regression model for each protein that included all genome-wide significant variants, anthropometrics, the 30 clinical chemistry parameters, sex, and visit. In the analysis, the genetic variants were combined as “genetic component” and all the anthropometric and clinical chemistry variables were combined as “environmental component.” A summary of the analysis across all 794 analyzed plasma proteins (Fig. 5a) shows that the influence of genetics and environment on blood protein level variability varies considerably. Limited longitudinal effects were found in the variability of proteins with genetic associations with an average contribution of 2%, suggesting that the protein levels associated with genetics are relatively stable throughout the 2-year study period. Out of the 107 proteins with significant pQTL associations, 56 proteins have at least a 50% contribution from genetics (Fig. 5b). The FOLR3 protein is the most affected protein with 98% of the blood protein level variance explained by genetics. Membrane metalloendopeptidase (MME), which is involved in the destruction of opioid peptides by cleavage of a Gly-Phe bond [41], is an example of a protein with the concentration levels in blood strongly associated with both genetic and environmental components, mainly due to the liver marker GGT (Additional file 1: Fig. S7A). Another example is protein carbonic anhydrase 5A (CA5A), which is a liver enriched gene [36], with the concentration levels mainly affected from genetics (60%) but also from ALAT (7%) (Additional file 1: Fig.S7B). The results demonstrate the importance of determining the underlying genetic make-up when analyzing individual differences in blood protein levels.

One hundred eighty-six proteins have at least a 10% contribution from a certain environmental component to the variability of the blood concentration levels (Additional file 1: Table S3). Among them, 63 proteins are associated with kidney function, 33 proteins are associated with lipid profile, 32 proteins are associated with body composition, 21 proteins are associated with leukocytes, and 42 proteins are associated with other clinical parameters. The top 30 most significant proteins associated with environmental components and with no genetic component are highlighted in Fig. 5c. A CCA [42] was also performed to investigate the associations of protein profiles with anthropometric and clinical chemistry variables. Associations of all analyzed samples (*n* = 540), together with proteins and clinical or anthropometric variables, were presented in the triplot (Fig. 5d). The CCA (Fig. 5d) predicts the effects of the plasma protein data and clinical parameters on sample levels and highlights that LEP is highly positively correlated with body fat and negatively correlated with bone mass and muscle mass. As an example, N-terminal pro-brain natriuretic peptide (NT-proBNP) and natriuretic peptide (BNP) were highly correlated with the NTproBNP levels in clinical chemistry, consistent with linear regression analysis result in Fig. 5b. Sex differences can be also observed, for example with higher skeletal muscle mass and Hb levels in males and higher body fat mass and HDL levels in females. Glycoprotein hormones, alpha polypeptide (CGA) which is a placenta-enriched protein, showed the largest sex difference with high levels of concentration in female samples. Prokineticin 1 (PROK1), on the other hand, showed higher concentration levels in male samples. The majority of proteins with significant pQTL variants were as expected shown not significantly associated with clinical or anthropometric variables but are located in the center of the plot.

### Changes due to environmental factors

**Fig. 6:**
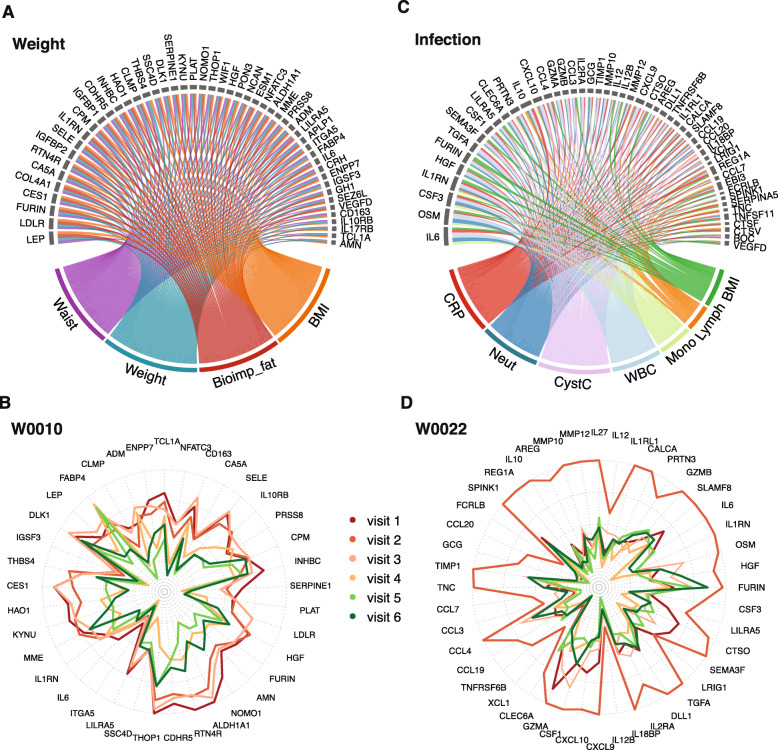
Dynamic molecular profiling changes and impact on weight loss and infection. **a** Chord diagram of the 50 most significant proteins related to body composition (bioimpedance fat, bioimpedance muscle, bioimpedance bone, weight, waist and BMI). The size of the link is defined as the absolute value of coefficient of the corresponding effect, and proteins are sorted based on the coefficient calculated using mixed-effect modeling. **b** A radar plot showing the protein profiles of the 37 most significant proteins positively related to body composition for the subject W0010 who had a 15.4 kg weight loss in 3 months between 3 and 4 and a total weight loss of 16.6 kg during the 2 years. **c** Chord diagram of the 50 most significant proteins related to CRP and including top six other parameters with significant effect to the same proteins. The size of the link is defined as the absolute value of coefficient of the corresponding effect and proteins are sorted based on the coefficient calculated using mixed-effect modeling. **d** Radar plots of the positively correlated proteins (*n* = 44) showing the relative abundance level in subject W0022 who had an increased CRP of 79 between visit 1 and 2

To investigate the effect of life style changes and in particular weight changes on the proteome, we focused on the mixed effect modeling results for weight-related anthropometrics (weight, waist, BMI, and bioimpedance fat) and obtained a list of the top 50 most significant proteins. The resulting connections between proteins and weight-related parameters are visualized as a chord diagram plot (Fig. 6a), and the protein profiling data was used to perform hierarchical clustering of the 50 proteins based on their concentration levels across the six visits (Additional file 1: Fig. S8A). We assessed the changes in plasma protein profiles before and after weight loss, exemplified by the participant W0010 who showed a large weight loss between visit three (120.5 kg) and visit four (104.7 kg), but started a change in lifestyle already after visit two. The protein levels in each of the six visits are visualized for all proteins with positive (*n* = 37) (Fig. 6b) or negative (*n* = 13) (Additional file 1: Fig. S8B) correlations with weight-related anthropometrics, respectively, highlighting the large changes between visits three and four for many of these proteins. We also compared the ratio of the complete set of plasma protein profiles between visits two and four (Additional file 1: Fig. S8C) to highlight the most altered proteins for this individual, and here, we see that the growth hormone protein (GH) had the largest change over all.

Finally, to get a comprehensive mapping of the proteome changes during an infection, we focused the multivariate analysis on the plasma protein profiles and their relationship with the CRP (Fig. 6c**)**. Based on linear mixed effect modeling results, the top 50 most highly associated proteins with CRP are visualized in Fig. 6c, and the circular dendrogram (Additional file 1: Fig. S8D) shows the relationship based on correlation of protein profiles between these mainly inflammatory and immunity-related proteins. An analysis of the same proteins in the individual with a serious infection at visit two shows an increase of a whole cascade of inflammatory-related proteins upon infection with the positively correlated proteins (*n* = 44) in Fig. 6d, with the largest change of many of the proteins in visit two. The small number of negatively correlated proteins (*n* = 6) is shown for the same individual in Additional file 1: Fig. S8E. The top driving proteins mainly include cytokines IL1RL1, IL1RN, IL27, IL12, IL6, and IL10; chemokines CCL3, CCL4, CCL7, CCL20, CXCL9, and CXCL10; also tumor necrosis factor TNFRSF6B, DLL1, and XCL1; a peptidase MMP12; and the growth factor TGFA. Additional file 1: Fig. S8F shows the log2-ratio between visit two and visit one for all proteins in the same individual, which clearly shows that IL17C, GCG, and REG1A have the largest increase in concentration and at the other end, ALDH3A1 decreased the most.

## Discussion

Here, we have combined whole-genome sequencing, multiplex protein profiling, and extensive clinical phenotyping to determine genetic associations related to the variability of blood levels of proteins based on a longitudinal wellness study of healthy individuals with repeated sampling. We present, for the first time, a longitudinal study in which a quantitative and sensitive protein extension assay has been combined with whole-genome sequencing. By combining eleven protein assay panels covering inflammation, cardiovascular disease, neurological disease, organ damage, and cancer, close to 800 proteins were studied with multiple sampling of all individuals.

Most of the proteins were stable over time with limited effect of longitudinal variation, with larger inter-individual variation as compared to the intra-individual variation. The use of whole genome sequencing allowed us to identify many more genetic variants influencing blood protein levels, and approximately half of the pQTLs found have not been reported earlier. The study confirms that the human blood level of many proteins during adult life is determined by genetics and that in clinically healthy study participants, these levels were stable during the 2-year study period.

The protein profile variability as a consequence of environmental factors was also analyzed. An interesting finding is the sex differences in both proteome and clinical chemistry, which is important for our understanding of both health and disease to avoid sex biased interpretations. In total, 186 proteins have at least a 10% contribution to the variability of the blood concentration levels from a certain environmental component measured in the study. Leptin (LEP), a key player in the regulation of energy balance and body weight control, is the most significant protein associated with known environmental factors, with more than 75% of the variance explained by the body composition. Another well-known example is low-density lipoprotein receptor (LDLR), the major cholesterol-carrying lipoprotein of plasma, which showed high associations with lipid profiles. Several immune-related molecules were also identified with high correlations with acute phase or leukocytes, including oncostatin M (OSM), interleukin 6 (IL6), interleukin 1 receptor antagonist (IL1RN), and matrix metallopeptidase 9 (MMP9), which is in line with the previous report that variation in the human immune system is largely associated with non-heritable factors [43].

The analysis of the individual molecular profiles revealed large effects on several proteins as a consequence of weight loss and infection. This analysis shows that weight loss resulted in a remodeling of many proteins, primarily involved in energy balance, insulin sensitivity, and adiposity-related processes with the main proteins driving this effect being LEP, LDLR, FURIN, and carboxylesterase 1 (CES1). Note that the changed blood levels for these proteins remain also during visit 5 and 6. The data confirms that leptin levels are associated with anthropometrics and ApoB/ApoA1 ratio and thus serves as a key metabolic marker [44]. The IGF binding proteins 1 and 2 are also among the most co-varying proteins, and these proteins are known to be associated with obesity and weight disorders [45]. The PON3 protein belongs to the paraoxonase family and is known to bind with HDL having antioxidant properties by rapidly hydrolyzing lactones to prevent LDL oxidation [46]. Our study also shows that weight loss results in a distinct molecular response of the PON3 protein.

The correlation analysis related to infection revealed the relationship between CRP-proteins and a number of other parameters, such as the biomarkers of kidney function cystatin C as well as the total leukocyte particle concentration (LPC). The elevated kidney biomarkers could reflect a transient reduction in kidney function often seen during infection. A whole cascade of inflammatory related proteins was shown to be affected to trigger and maintain the inflammatory and immunological responses related to infection. Interestingly, our data confirmed the relationship between CRP and IL-6, but the results also give a broader view of the cytokines landscape that are significantly connected with CRP. This may lead to a better stratified molecular understanding of the biological mechanisms underlying CRP effects in inflammation and related diseases.

Several important conclusions can be drawn with relevance for precision medicine efforts. First, the study suggests that protein levels throughout adult life are affected by precise genetic variants for more than 100 proteins analyzed here. Genetics should therefore be considered when assessing an individual’s protein levels. As an example, the FOLR3 protein, with a remarkable 98% contribution to plasma levels from genetics, has higher plasma levels for both the homozygote and heterozygote variants of the corresponding gene. In contrast, the heterozygote individuals for the gene coding for PDGFRB has intermediate protein levels for the heterozygote variant as compared to the two homozygote individuals. Second, the protein levels determined by genetics are stable throughout the study period suggesting that these blood levels are indeed stable throughout adult life. Third, several proteins with strong association with known clinical parameters have been identified, opening up for validation studies in large cohort to establish if these protein markers can be used as complement to the assays already used in the clinic. Fourth, the profound changes by environmental factors are also demonstrated, here exemplified by the dramatic changes in global protein profiles upon weight loss and infection, and thus, we have identified proteins to be targeted for dedicated studies involving larger cohorts to validate their clinical usefulness.

## Conclusions

In summary, we show that the human blood level of many proteins during adult life is to a large extent affected by genetics, which is important for precision medicine efforts aimed to understand the individual differences of protein levels and the relationship between plasma proteome profiles and human biology and disease.

## Supplementary information

**Additional file 1: Fig. S1**. Reference sample normalization. **Fig. S2**. Technical variation for proteins with data from multiple Olink panels. **Fig. S3**. Hierarchical clustering of 540 samples. **Fig. S4**. Variation of plasma protein profiling of the ten outlier subjects from the clustering. **Fig. S5**. Variation of weight and infection levels during two year. **Fig. S6**. Significant levels of pQTL variants and the associated proteins. **Fig. S7**. Examples of proteins with both genetic and environmental effects. **Fig. S8**. Dynamic molecular profiling changes and impact on weight loss and infection. **Table S1**. Description of the anthropometric and clinical chemistry parameters **Table S2.** Variability of the plasma proteins. **Table S3**. A list of 186 proteins with at least a 10% contribution from a certain environmental component.

**Additional file 2: Table S4**. Summary of the identified independent pQTLs. **Table S5**. Overlap of cis-eQTLs with cis-pQTLs. **Table S6.** Disease association with the pQTLs.

## Data Availability

All summary statistics and association data are available in the supplementary material. The participant-level genotype and phenotype datasets of S3WP program have been deposited with the Swedish National Data Service (http://snd.gu.se, a data repository certified by Core Trust Seal) [19]. Due to patient consent and confidentiality agreements, the dataset can only be made available for validation purposes by contacting snd@snd.gu.se. Data access will be evaluated according to Swedish legislation. Data access for research related questions in the S3WP program can be made available by contacting the corresponding author.

## Abbreviations

ALAT: Alanine aminotransferase
ANOVA: Analysis of variance
ApoA1: Apolipoprotein A1
ApoB: Apolipoprotein B
BMI: Body mass index
CCA: Canonical correspondence analysis
CGA: Glycoprotein hormones, alpha polypeptide
CV: Coefficient of variation
CRP: C-reactive protein
GGT: Gamma-glutamyltransferase
GWAS: Genome-wide association study
Hb: Hemoglobin
HbA1c: Hemoglobin A1c
hsCRP: High sensitive C-reactive protein
HDL: High-density lipoprotein
IBS: Identity-by-state
IQR: Interquartile range
LEP: Leptin
LD: Linkage disequilibrium
LDL: Low-density lipoprotein
LDLR: Low-density lipoprotein receptor
NT-proBNP: N-Terminal pro-brain natriuretic peptide
MEP1B: Meprin A subunit beta
MDS: Multidimensional scaling
NPX: Normalized protein expression
PDGFRB: Platelet-derived growth factor receptor beta
PEA: Protein extension analysis
pQTL: Protein quantitative trait locus
QC: Quality control
SCAPIS: Swedish CArdioPulmonary bioImage Study
S3WP: Swedish SciLifeLab SCAPIS Wellness Profiling
TG: Triglycerides
TNT: Troponin T
