# Supplementary material for: Whole-genome sequence association analysis of blood proteins in a longitudinal wellness cohort
Source: Genome Med. 2020 Jun 23;12:53. doi: 10.1186/s13073-020-00755-0 (PMC7310558; doi:10.1186/s13073-020-00755-0)
Supplement: Supplementary file 1 — Additional file 1: Fig. S1. Reference sample normalization. Fig. S2. Technical variation for proteins with data from multiple Olink panels. Fig. S3. Hierarchical clustering of 540 samples. Fig. S4. Variation of plasma protein profiling of the ten outlier subjects from the clustering. Fig. S5. Variation of weight and infection levels during two year. Fig. S6. Significant levels of pQTL variants and the associated proteins. Fig. S7. Examples of proteins with both genetic and environmental effects. Fig. S8. Dynamic molecular profiling changes and impact on weight loss and infection. Table S1. Description of the anthropometric and clinical chemistry parameters Table S2. Variability of the plasma proteins. Table S3. A list of 186 proteins with at least a 10% contribution from a certain environmental component. [file 13073_2020_755_MOESM1_ESM.docx]

**Fig S1**


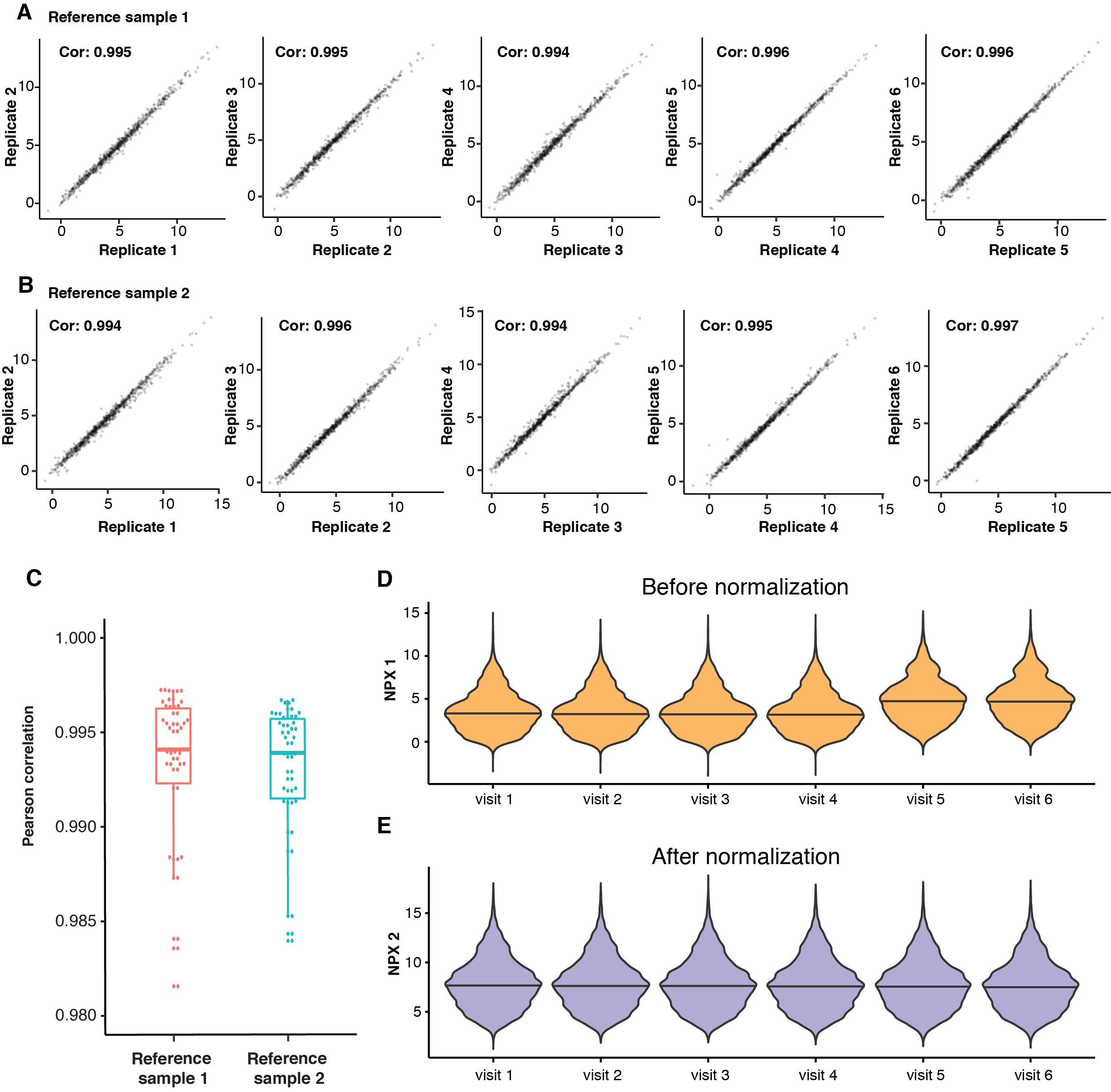


**Fig S1. Reference sample normalization.** (A-B) Scatter plots showing pairwise comparisons of the replicates within two reference samples, which have been run in different plates as bridge controls. (C) Overall Pearson correlation between reference samples used for normalization. Violin plots showing the distribution of protein levels (D) before normalization and (E) after normalization across six visits.

**Fig S2**


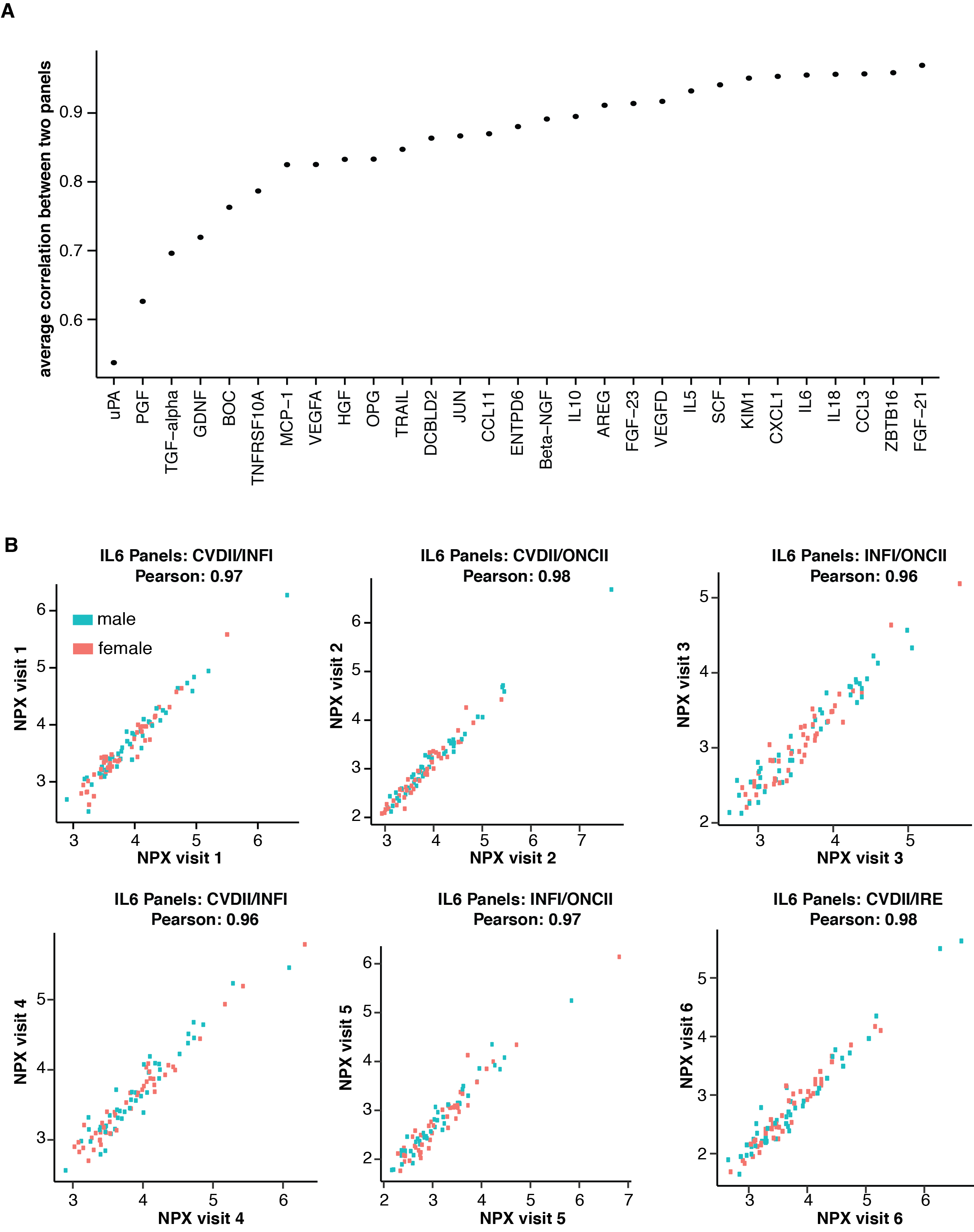


**Fig S2.** **Technical variation for proteins with data from multiple Olink panels.** (a) The average correlation between the same visit in different panels for 29 proteins run in multiple panels. (b) Scatterplots showing examples of the high correlation between data from different panels and the same visit for the IL6 protein, which has been run in four different panels (CVDII, INFI, ONCII and IRE).

**Fig S3**


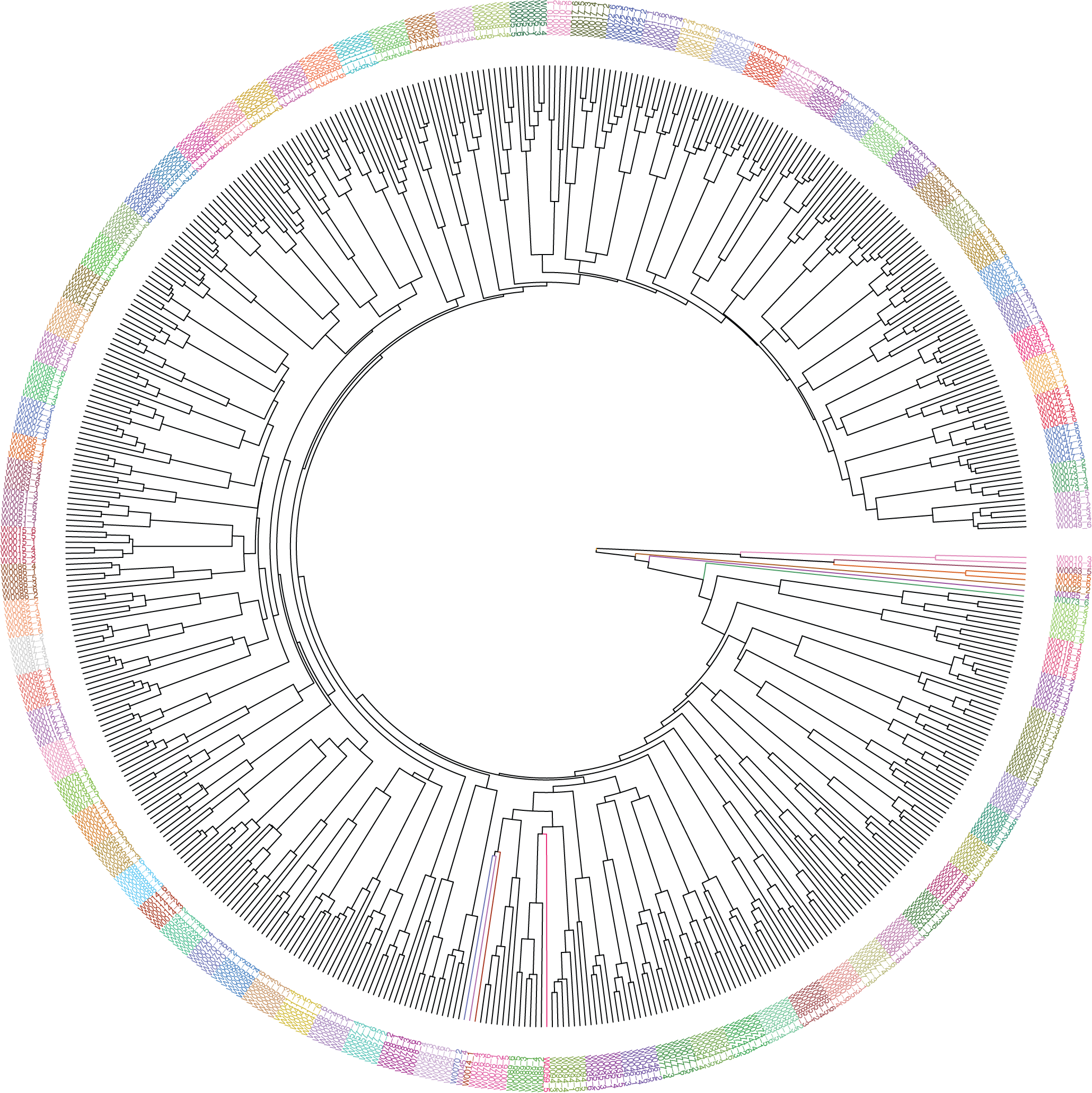


**Fig S3.** **Hierarchical clustering of 540 samples.** Hierarchical clustering based on pair-wise Pearson correlation of the protein expression in all 540 samples is shown with labels color coded by subject. The most distinct samples that deviate from other samples within subjects are highlighted.

**Fig S4**

**
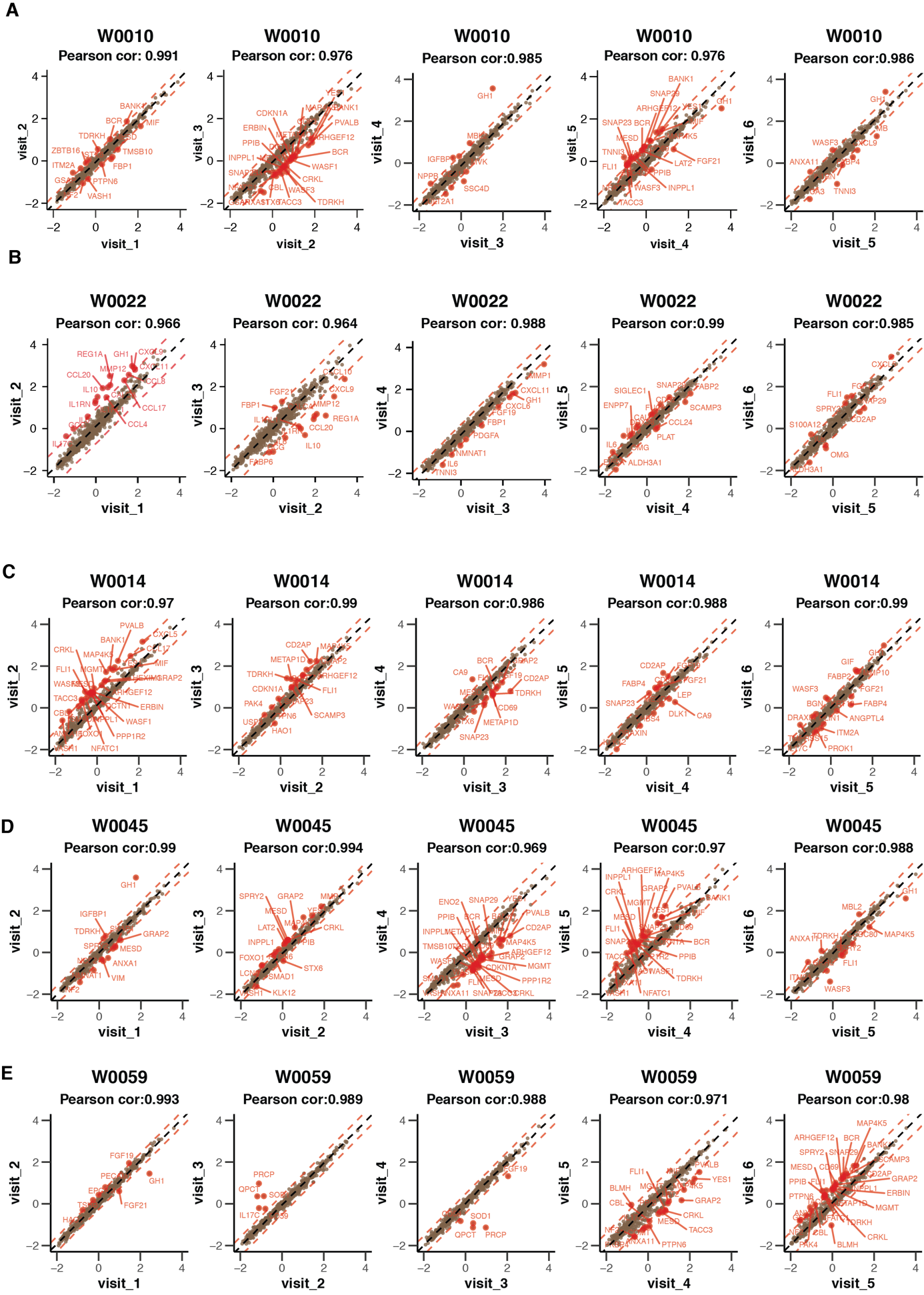
**


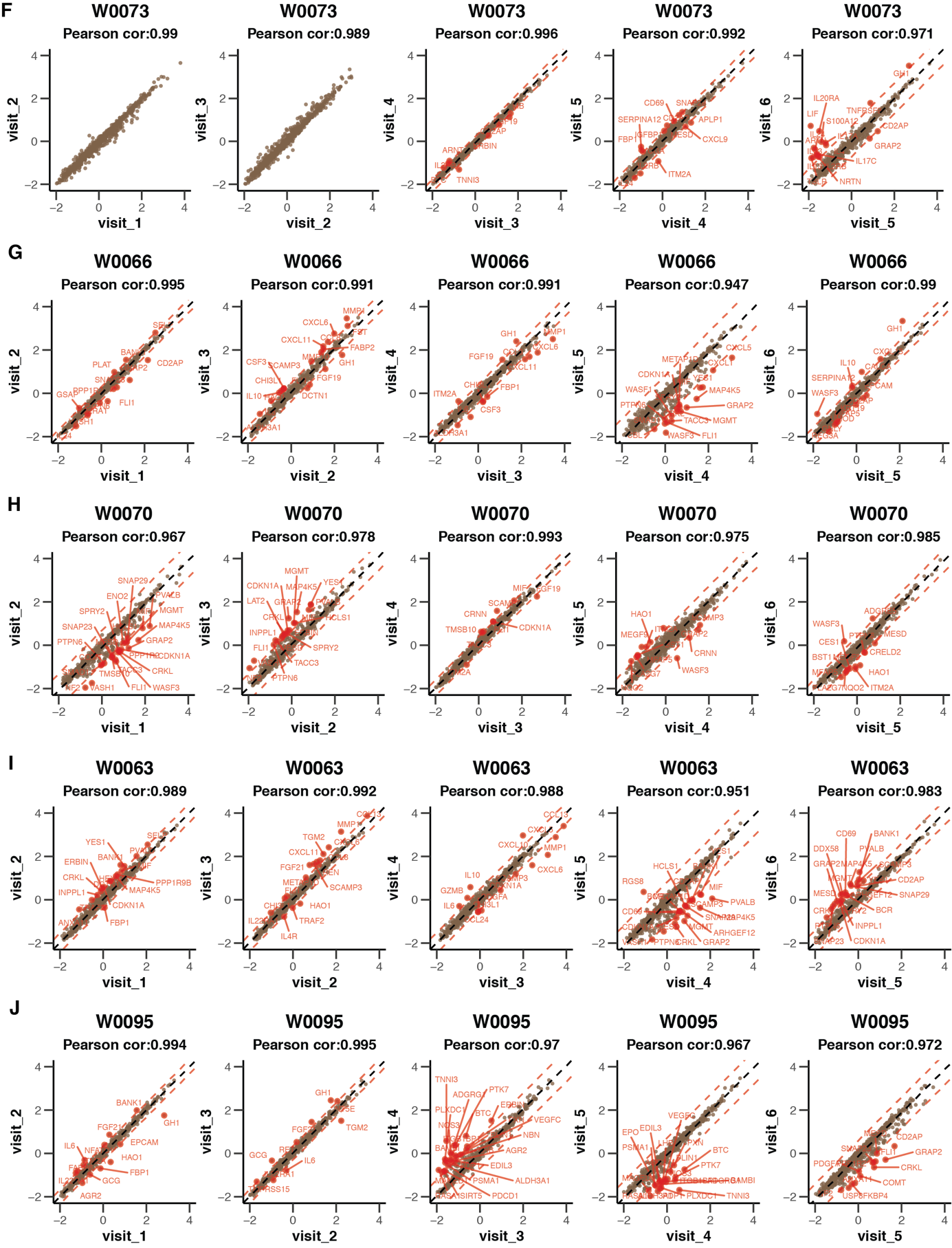


**Fig S4. Variation of plasma protein profiling of the ten outlier subjects from the clustering.** Scatter plots of differential protein levels across six visits in the ten outlier subjects from the hierarchical clustering.

**Fig S5**

**
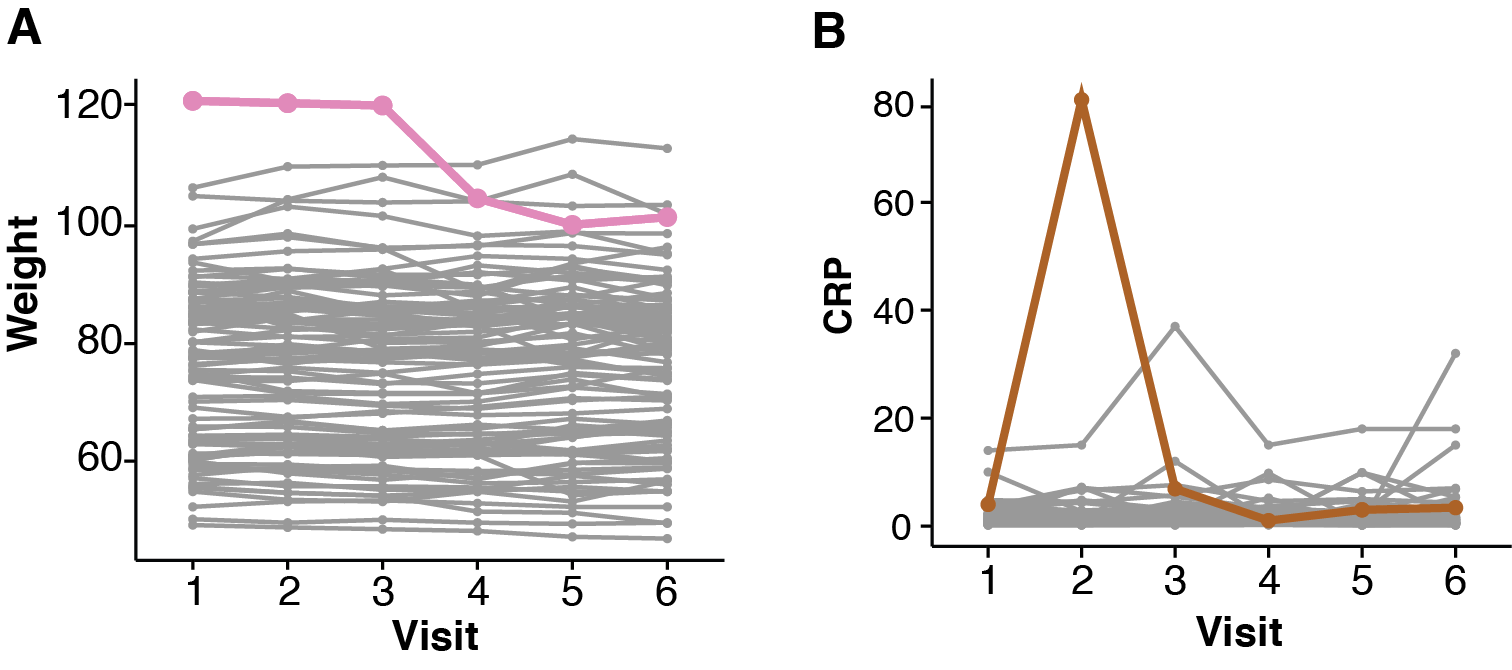
**

**Fig S5. Variation of weight and infection levels during two year.** (A) The longitudinal weight distribution across the six visits with subject W0010 highlighted in pink. (B) The longitudinal C-reactive protein (CRP) distribution across the six visits with subject W0022 highlighted in brown.

**Fig S6**

**
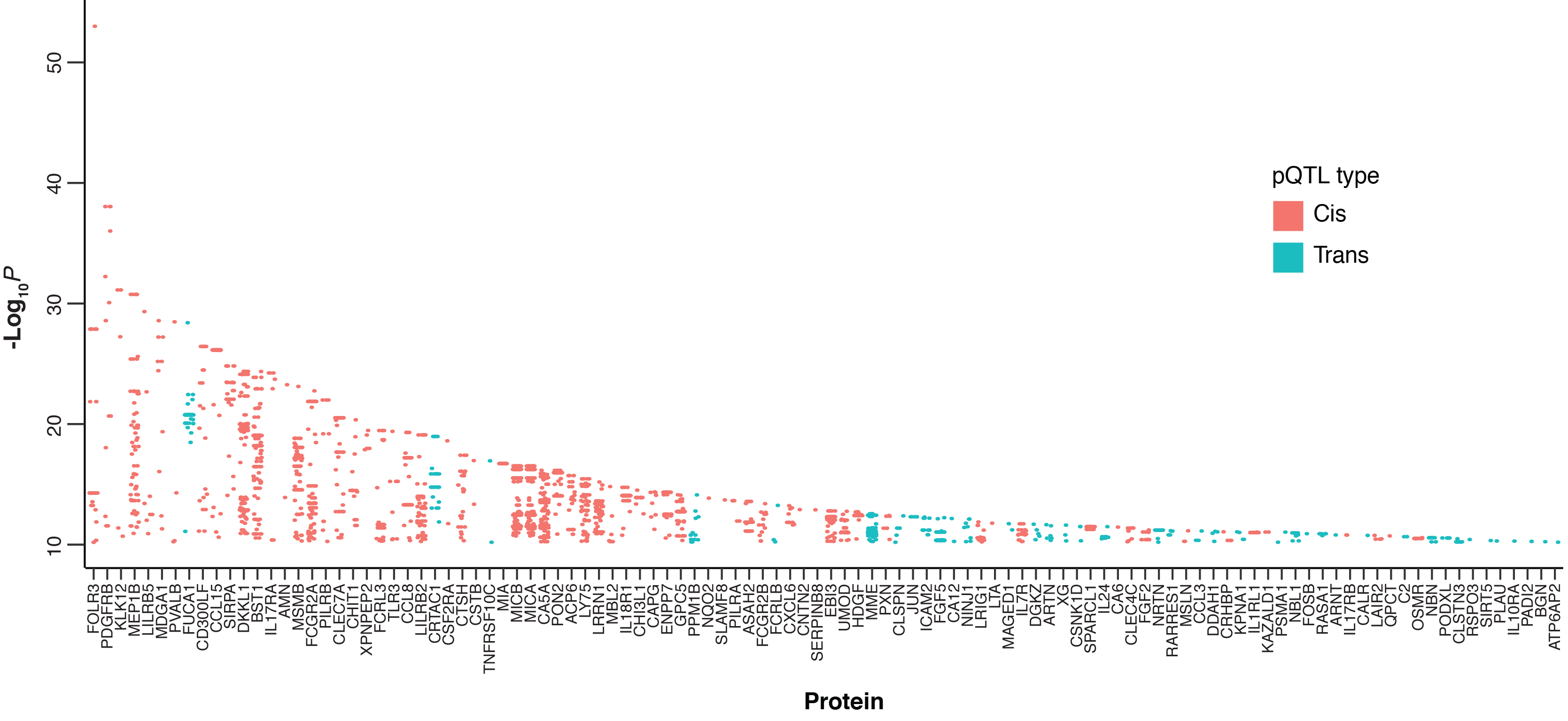
**

**Fig S6. Significant levels of pQTL variants and the associated proteins.** Scatter plot showing the significant levels of pQTLs and the associated proteins, colored by cis- and trans-pQTLs.

**Fig S7**

**
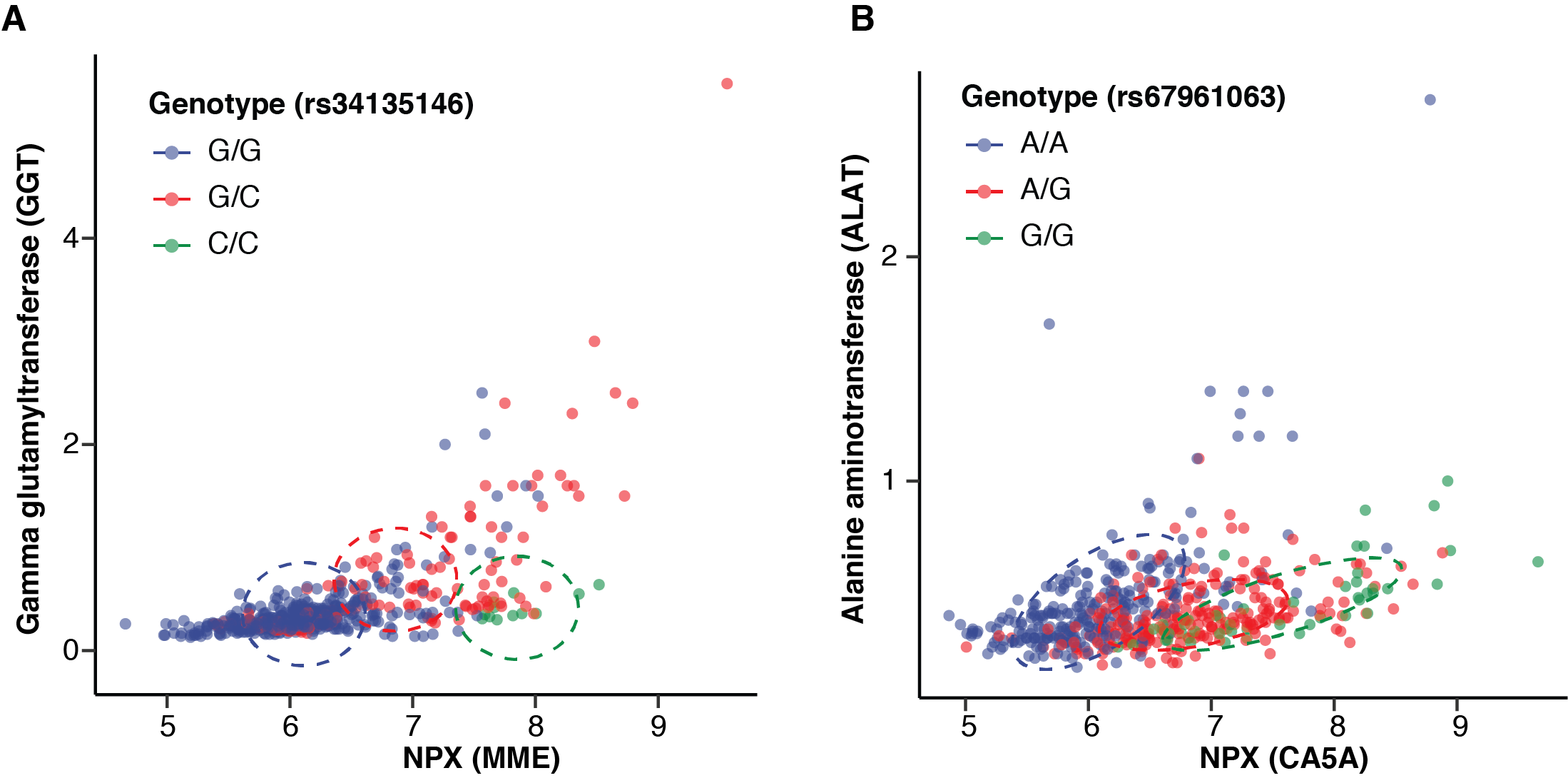
**

**Fig S7. Examples of proteins with both genetic and environmental effects.** Scatter plots showing the relationships between (A) the levels of gamma glutamyltransferase (GGT) and blood levels of protein MME, (B) the levels of alanine aminotransferase (ALAT) and blood levels of protein CA5A, colored by the genotypes of individuals.

**Fig S8**

**
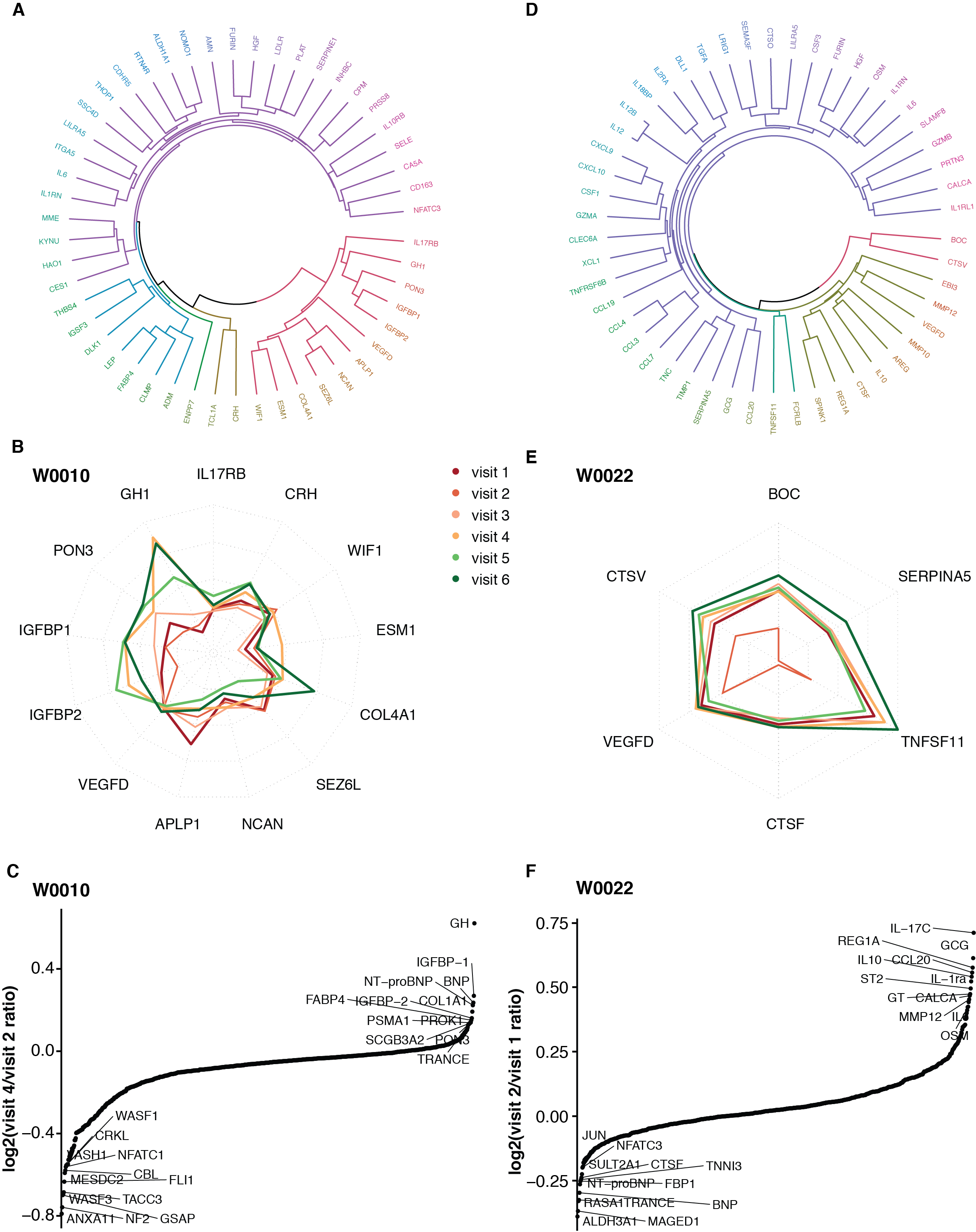
**

**Fig S8. Dynamic molecular profiling changes and impact on weight loss and infection.** (A) Circular dendrogram based on the pairwise correlation between the 50 most significant proteins related to anthropometrics (bioimpedance fat, weight, waist and BMI). (B) Radar plot showing the protein profiles of the negatively correlated proteins (n=13) from (A) for the subject W0010 who had a 15.4 kg weight loss in three months between 3 and 4 and a total weight loss of 16.6 kg during the first year. (C) Log-2 ratio of the protein levels between visit 4 and 2 for subject W0010. (D) Circular dendrogram based on the pairwise correlation between the 50 most significant proteins related to CRP. (E) Radar plot showing the protein profiles of the negatively correlated proteins (n=7) from (D) for the subject W0022 with a high CRP value in visit 2. (E) Log-2 ratio of the protein levels between visit 1 and 2 for subject W0022.

**Table S1:** **Description of the clinical chemistry and anthropometric variables.**

| Clinical/ Anthropometric variable | Full name (unit) | Biomarker group |
| --- | --- | --- |
| Age | Age at visit (years) | Age |
| Weight | Body weight (kg) | Body composition |
| Height | Height (cm) | Body composition |
| BMI | Body_mass_index (kg/m2) | Body composition |
| Waist | Waist circumference (cm) | Body composition |
| Hip | Hip circumference (cm) | Body composition |
| WHR | Weight to Height ratio () | Body composition |
| Bioimp_fat | Body fat content (%) | Body composition |
| Bioimp_muscle | Body muscle content (%) | Body composition |
| Bioimp_bone | Body bone content (%) | Body composition |
| SBP | Systolic blood pressure (mmHg) | Blood pressure |
| DBP | Diastolic blood pressure (mmHg) | Blood pressure |
| Gluc | Glucose (mmol/L) | Glucose homeostasis |
| HbA1c | Hemoglobin A1c (mmol/mol) | Glucose homeostasis |
| Chol | Total cholesterol (mmol/L) | Lipid profile |
| LDL | Low density lipoprotein cholesterol (mmol/L) | Lipid profile |
| HDL | High density lipoprotein cholesterol (mmol/L) | Lipid profile |
| TG | Triglycerides (mmol/L) | Lipid profile |
| ApoA1 | Apolipoprotein A1 (g/L) | Lipid profile |
| ApoB | Apolipoprotein B (g/L) | Lipid profile |
| ApoB/ApoA1 | Apolipoprotein B / Apolipoprotein A1 ratio | Lipid profile |
| ALAT | Alanine aminotransferase (micromol/L) | Liver |
| GGT | Gamma glutamyltransferase (microkat/L) | Liver |
| CRP | C-reactive protein, high sensitivity (mg/L) | Acute phase |
| Urate | Urate (micromol/L) | Urate |
| Crea | Creatinine (micromol/L) | Kidney |
| CystC | Cystatin C (mg/L) | Kidney |
| NTproBNP | N-terminal pro b-type natriuretic peptide (ng/L) | Heart |
| TNT | Troponin T, high sensitivity (g/L) | Heart |
| Hb | Hemoglobin (g/L) | Erythrocytes |
| Hct | Hematocrit (erythrocyte volume fraction) (L/L) | Erythrocytes |
| MCH | Mean corpuscular hemoglobin (pg) | Erythrocytes |
| MCHC | Mean corpuscular hemoglobin conc. (g/L) | Erythrocytes |
| MCV | Mean corpuscular volume (fL) | Erythrocytes |
| RBC | Red blood cell count (x10*12/L) | Erythrocytes |
| Plt | Platelet count (x10*9/L) | Platelets |
| WBC | White blood cell count (x10*9/L) | Leukocytes |
| Neut | Neutrophil count (x10*9/L) | Leukocytes |
| Lymph | Lymphocyte count (x10*9/L) | Leukocytes |
| Mono | Monocyte count (x10*9/L) | Leukocytes |
| Eos | Eosinophil count (x10*9/L) | Leukocytes |
| Baso | Basophil count (x10*9/L) | Leukocytes |

**Table S2:** **Variability of all proteins and their corresponding intra- and inter-individual CV**

| Protein | Description | Median expression level | IQR (Log2 FC) | Intra-ind CV | Inter-ind CV | FDA drug target |
| --- | --- | --- | --- | --- | --- | --- |
| KLK12 | kallikrein related peptidase 12 | 5.279 | 0.325 | 0.063 | 0.182 | no |
| GH2 | growth hormone 2 | 5.340 | 0.322 | 0.056 | 0.310 | no |
| CRX | cone-rod homeobox | 4.485 | 0.319 | 0.086 | 0.282 | no |
| SSC4D | scavenger receptor cysteine rich family member with 4 domains | 5.888 | 0.284 | 0.056 | 0.221 | no |
| CGA | glycoprotein hormones, alpha polypeptide | 8.021 | 0.284 | 0.041 | 0.160 | no |
| JUN | Jun proto-oncogene, AP-1 transcription factor subunit | 2.696 | 0.281 | 0.137 | 0.272 | yes |
| MEP1B | meprin A subunit beta | 7.207 | 0.251 | 0.044 | 0.174 | no |
| PROK1 | prokineticin 1 | 5.977 | 0.226 | 0.037 | 0.127 | no |
| GH1 | growth hormone 1 | 14.263 | 0.224 | 0.072 | 0.139 | no |
| HAO1 | hydroxyacid oxidase 1 | 7.424 | 0.219 | 0.083 | 0.151 | no |
| LHB | luteinizing hormone beta polypeptide | 5.710 | 0.216 | 0.044 | 0.168 | no |
| NF2 | neurofibromin 2 | 4.918 | 0.215 | 0.126 | 0.157 | no |
| SULT2A1 | sulfotransferase family 2A member 1 | 3.491 | 0.200 | 0.122 | 0.141 | no |
| VASH1 | vasohibin 1 | 5.723 | 0.197 | 0.126 | 0.153 | no |
| KPNA1 | karyopherin subunit alpha 1 | 3.616 | 0.196 | 0.132 | 0.130 | no |
| NPPB | natriuretic peptide B | 4.556 | 0.194 | 0.064 | 0.149 | no |
| CEACAM5 | carcinoembryonic antigen related cell adhesion molecule 5 | 4.662 | 0.193 | 0.058 | 0.162 | no |
| CD177 | CD177 molecule | 7.939 | 0.193 | 0.030 | 0.176 | no |
| EPCAM | epithelial cell adhesion molecule | 9.006 | 0.185 | 0.045 | 0.123 | yes |
| SERPINA12 | serpin family A member 12 | 6.805 | 0.185 | 0.052 | 0.158 | no |
| ANXA11 | annexin A11 | 6.329 | 0.184 | 0.107 | 0.160 | no |
| AGR2 | anterior gradient 2, protein disulphide isomerase family member | 5.103 | 0.181 | 0.073 | 0.142 | no |
| FLI1 | Fli-1 proto-oncogene, ETS transcription factor | 8.155 | 0.178 | 0.099 | 0.127 | no |
| GHRL | ghrelin and obestatin prepropeptide | 6.015 | 0.177 | 0.044 | 0.130 | no |
| FBP1 | fructose-bisphosphatase 1 | 7.196 | 0.175 | 0.103 | 0.118 | no |
| RGS8 | regulator of G protein signaling 8 | 2.925 | 0.174 | 0.053 | 0.177 | no |
| PTN | pleiotrophin | 5.719 | 0.173 | 0.044 | 0.125 | no |
| RASA1 | RAS p21 protein activator 1 | 2.761 | 0.173 | 0.079 | 0.283 | no |
| TNNI3 | troponin I3, cardiac type | 4.281 | 0.170 | 0.092 | 0.173 | no |
| IGFBP1 | insulin like growth factor binding protein 1 | 8.596 | 0.169 | 0.052 | 0.118 | no |
| NA | NA | 7.543 | 0.168 | 0.061 | 0.122 | no |
| CA5A | carbonic anhydrase 5A | 6.449 | 0.166 | 0.056 | 0.118 | no |
| SCGB3A2 | secretoglobin family 3A member 2 | 5.200 | 0.163 | 0.053 | 0.147 | no |
| WASF3 | WAS protein family member 3 | 8.264 | 0.163 | 0.100 | 0.124 | no |
| PVALB | parvalbumin | 12.337 | 0.163 | 0.051 | 0.135 | no |
| ENPP7 | ectonucleotide pyrophosphatase/phosphodiesterase 7 | 7.479 | 0.161 | 0.040 | 0.107 | no |
| GSAP | gamma-secretase activating protein | 5.368 | 0.161 | 0.084 | 0.160 | no |
| LEP | leptin | 9.883 | 0.160 | 0.033 | 0.113 | no |
| HDGF | heparin binding growth factor | 5.513 | 0.160 | 0.081 | 0.120 | no |
| CRH | corticotropin releasing hormone | 5.921 | 0.160 | 0.045 | 0.111 | no |
| FGF2 | fibroblast growth factor 2 | 5.224 | 0.159 | 0.061 | 0.121 | yes |
| BTC | betacellulin | 5.651 | 0.158 | 0.069 | 0.123 | no |
| CCL24 | C-C motif chemokine ligand 24 | 7.978 | 0.157 | 0.034 | 0.109 | no |
| TCL1A | T cell leukemia/lymphoma 1A | 8.631 | 0.157 | 0.039 | 0.117 | no |
| STX6 | syntaxin 6 | 6.923 | 0.156 | 0.085 | 0.113 | no |
| ALDH3A1 | aldehyde dehydrogenase 3 family member A1 | 4.231 | 0.153 | 0.071 | 0.139 | no |
| MSMB | microseminoprotein beta | 6.132 | 0.151 | 0.034 | 0.120 | no |
| TDRKH | tudor and KH domain containing | 7.777 | 0.149 | 0.076 | 0.121 | no |
| AMN | amnion associated transmembrane protein | 6.599 | 0.149 | 0.025 | 0.099 | no |
| SFTPD | surfactant protein D | 6.069 | 0.148 | 0.043 | 0.106 | no |
| OMG | oligodendrocyte myelin glycoprotein | 5.936 | 0.145 | 0.050 | 0.100 | no |
| CRTAC1 | cartilage acidic protein 1 | 5.687 | 0.145 | 0.039 | 0.111 | no |
| CA6 | carbonic anhydrase 6 | 5.288 | 0.144 | 0.038 | 0.118 | no |
| TMPRSS15 | transmembrane serine protease 15 | 4.194 | 0.144 | 0.054 | 0.106 | no |
| ZBTB16 | zinc finger and BTB domain containing 16 | 6.589 | 0.142 | 0.069 | 0.113 | no |
| CDKN1A | cyclin dependent kinase inhibitor 1A | 9.033 | 0.142 | 0.082 | 0.103 | no |
| SMAD1 | SMAD family member 1 | 7.062 | 0.142 | 0.087 | 0.109 | no |
| IL4 | interleukin 4 | 3.862 | 0.142 | 0.084 | 0.201 | no |
| ITM2A | integral membrane protein 2A | 5.400 | 0.141 | 0.082 | 0.102 | no |
| IL24 | interleukin 24 | 4.441 | 0.140 | 0.086 | 0.165 | no |
| LAIR2 | leukocyte associated immunoglobulin like receptor 2 | 7.259 | 0.140 | 0.022 | 0.159 | no |
| TREM1 | triggering receptor expressed on myeloid cells 1 | 2.962 | 0.139 | 0.050 | 0.107 | no |
| OSM | oncostatin M | 6.396 | 0.139 | 0.054 | 0.102 | no |
| LILRB5 | leukocyte immunoglobulin like receptor B5 | 7.494 | 0.139 | 0.027 | 0.116 | no |
| CBL | Cbl proto-oncogene | 7.023 | 0.138 | 0.082 | 0.109 | no |
| CHIT1 | chitinase 1 | 7.856 | 0.137 | 0.026 | 0.170 | no |
| PSMA1 | proteasome subunit alpha 1 | 4.156 | 0.137 | 0.038 | 0.157 | no |
| FKBP4 | FK506 binding protein 4 | 5.944 | 0.137 | 0.088 | 0.098 | no |
| IL7R | interleukin 7 receptor | 4.557 | 0.136 | 0.052 | 0.099 | no |
| FGF21 | fibroblast growth factor 21 | 9.926 | 0.136 | 0.057 | 0.100 | no |
| IL6 | interleukin 6 | 5.706 | 0.134 | 0.056 | 0.108 | yes |
| MME | membrane metalloendopeptidase | 6.167 | 0.134 | 0.034 | 0.116 | yes |
| TACC3 | transforming acidic coiled-coil containing protein 3 | 8.155 | 0.131 | 0.080 | 0.113 | no |
| PAK4 | p21 (RAC1) activated kinase 4 | 6.295 | 0.131 | 0.074 | 0.089 | no |
| GRAP2 | GRB2-related adaptor protein 2 | 9.945 | 0.130 | 0.081 | 0.099 | no |
| PRKCQ | protein kinase C theta | 4.747 | 0.130 | 0.072 | 0.116 | no |
| TRIM5 | tripartite motif containing 5 | 6.425 | 0.130 | 0.065 | 0.094 | no |
| TSHB | thyroid stimulating hormone beta | 7.350 | 0.129 | 0.042 | 0.099 | no |
| CCL20 | C-C motif chemokine ligand 20 | 8.983 | 0.129 | 0.042 | 0.122 | no |
| NQO2 | N-ribosyldihydronicotinamide:quinone reductase 2 | 3.939 | 0.128 | 0.068 | 0.104 | no |
| RNASE3 | ribonuclease A family member 3 | 5.377 | 0.128 | 0.057 | 0.098 | no |
| CTSH | cathepsin H | 5.908 | 0.128 | 0.036 | 0.088 | no |
| ARNT | aryl hydrocarbon receptor nuclear translocator | 4.591 | 0.128 | 0.062 | 0.218 | no |
| LAT2 | linker for activation of T cells family member 2 | 8.427 | 0.127 | 0.065 | 0.089 | no |
| WASF1 | WAS protein family member 1 | 8.645 | 0.127 | 0.066 | 0.099 | no |
| COMT | catechol-O-methyltransferase | 7.032 | 0.126 | 0.075 | 0.089 | yes |
| FCGR2B | Fc fragment of IgG receptor IIb | 6.169 | 0.125 | 0.043 | 0.096 | yes |
| MBL2 | mannose binding lectin 2 | 12.302 | 0.125 | 0.024 | 0.099 | no |
| FCRLB | Fc receptor like B | 5.110 | 0.125 | 0.034 | 0.174 | no |
| DDX58 | DExD/H-box helicase 58 | 6.565 | 0.125 | 0.064 | 0.087 | no |
| TSLP | thymic stromal lymphopoietin | 3.464 | 0.124 | 0.083 | 0.105 | no |
| SCAMP3 | secretory carrier membrane protein 3 | 10.640 | 0.124 | 0.067 | 0.092 | no |
| LYPD1 | LY6/PLAUR domain containing 1 | 3.612 | 0.124 | 0.044 | 0.205 | no |
| EDAR | ectodysplasin A receptor | 6.890 | 0.124 | 0.053 | 0.104 | no |
| PDGFA | platelet derived growth factor subunit A | 6.927 | 0.124 | 0.073 | 0.098 | no |
| USP8 | ubiquitin specific peptidase 8 | 5.377 | 0.123 | 0.075 | 0.088 | no |
| CLEC4D | C-type lectin domain family 4 member D | 5.317 | 0.123 | 0.039 | 0.097 | no |
| NFATC1 | nuclear factor of activated T cells 1 | 7.716 | 0.123 | 0.069 | 0.094 | no |
| EIF5A | eukaryotic translation initiation factor 5A | 3.715 | 0.122 | 0.067 | 0.092 | no |
| PTPN6 | protein tyrosine phosphatase, non-receptor type 6 | 7.366 | 0.122 | 0.081 | 0.094 | no |
| VEGFC | vascular endothelial growth factor C | 4.645 | 0.121 | 0.053 | 0.121 | no |
| IL13 | interleukin 13 | 3.767 | 0.120 | 0.065 | 0.111 | no |
| BLVRB | biliverdin reductase B | 3.987 | 0.120 | 0.078 | 0.074 | yes |
| CES1 | carboxylesterase 1 | 5.154 | 0.119 | 0.053 | 0.103 | yes |
| MAGED1 | MAGE family member D1 | 3.328 | 0.119 | 0.075 | 0.108 | no |
| APP | amyloid beta precursor protein | 5.179 | 0.119 | 0.073 | 0.091 | yes |
| TNC | tenascin C | 5.216 | 0.118 | 0.038 | 0.085 | no |
| PRKRA | protein activator of interferon induced protein kinase EIF2AK2 | 4.917 | 0.118 | 0.062 | 0.093 | no |
| CRNN | cornulin | 8.676 | 0.118 | 0.030 | 0.093 | no |
| MESD | mesoderm development LRP chaperone | 8.695 | 0.118 | 0.075 | 0.103 | no |
| CD2AP | CD2 associated protein | 10.624 | 0.116 | 0.069 | 0.084 | no |
| EPO | erythropoietin | 4.337 | 0.116 | 0.045 | 0.149 | no |
| FABP6 | fatty acid binding protein 6 | 5.760 | 0.115 | 0.058 | 0.099 | no |
| S100A12 | S100 calcium binding protein A12 | 4.765 | 0.114 | 0.067 | 0.106 | no |
| IL22RA1 | interleukin 22 receptor subunit alpha 1 | 5.357 | 0.114 | 0.075 | 0.081 | no |
| CA9 | carbonic anhydrase 9 | 7.176 | 0.114 | 0.042 | 0.101 | no |
| TRAF2 | TNF receptor associated factor 2 | 7.188 | 0.114 | 0.069 | 0.080 | no |
| CRKL | CRK like proto-oncogene, adaptor protein | 8.824 | 0.114 | 0.079 | 0.101 | no |
| INPPL1 | inositol polyphosphate phosphatase like 1 | 8.461 | 0.113 | 0.070 | 0.089 | no |
| CHRDL2 | chordin like 2 | 5.906 | 0.113 | 0.040 | 0.091 | no |
| FABP9 | fatty acid binding protein 9 | 4.865 | 0.113 | 0.035 | 0.087 | no |
| MSLN | mesothelin | 7.150 | 0.112 | 0.022 | 0.084 | no |
| GCG | glucagon | 5.469 | 0.112 | 0.060 | 0.095 | no |
| IL5RA | interleukin 5 receptor subunit alpha | 6.694 | 0.112 | 0.024 | 0.083 | yes |
| DKKL1 | dickkopf like acrosomal protein 1 | 5.371 | 0.112 | 0.026 | 0.091 | no |
| GZMB | granzyme B | 6.067 | 0.112 | 0.049 | 0.097 | no |
| MICA/B | MHC class I polypeptide-related sequence A/B | 7.985 | 0.112 | 0.017 | 0.176 | no |
| PLAT | plasminogen activator, tissue type | 8.888 | 0.112 | 0.054 | 0.062 | yes |
| CALCA | calcitonin related polypeptide alpha | 8.558 | 0.112 | 0.030 | 0.091 | no |
| SPRY2 | sprouty RTK signaling antagonist 2 | 8.595 | 0.112 | 0.066 | 0.093 | no |
| CAPG | capping actin protein, gelsolin like | 6.171 | 0.111 | 0.028 | 0.083 | no |
| CHI3L1 | chitinase 3 like 1 | 6.449 | 0.111 | 0.044 | 0.105 | no |
| BCR | BCR, RhoGEF and GTPase activating protein | 9.505 | 0.111 | 0.064 | 0.086 | yes |
| LTBP2 | latent transforming growth factor beta binding protein 2 | 3.450 | 0.111 | 0.080 | 0.093 | no |
| LYAR | Ly1 antibody reactive | 3.144 | 0.110 | 0.062 | 0.114 | no |
| CA3 | carbonic anhydrase 3 | 3.634 | 0.110 | 0.056 | 0.082 | yes |
| PPY | pancreatic polypeptide | 11.804 | 0.109 | 0.031 | 0.087 | no |
| SNAP23 | synaptosome associated protein 23 | 8.167 | 0.109 | 0.074 | 0.094 | no |
| MDGA1 | MAM domain containing glycosylphosphatidylinositol anchor 1 | 9.105 | 0.109 | 0.020 | 0.076 | no |
| MAP4K5 | mitogen-activated protein kinase kinase kinase kinase 5 | 11.435 | 0.109 | 0.073 | 0.084 | no |
| ERBIN | erbb2 interacting protein | 8.670 | 0.109 | 0.067 | 0.084 | no |
| CRISP2 | cysteine rich secretory protein 2 | 10.298 | 0.108 | 0.018 | 0.083 | no |
| KRT19 | keratin 19 | 7.274 | 0.108 | 0.058 | 0.087 | no |
| METAP1D | methionyl aminopeptidase type 1D, mitochondrial | 9.070 | 0.107 | 0.061 | 0.088 | no |
| LDLR | low density lipoprotein receptor | 7.225 | 0.107 | 0.038 | 0.078 | no |
| MUC16 | mucin 16, cell surface associated | 7.802 | 0.106 | 0.027 | 0.088 | no |
| GAL | galanin and GMAP prepropeptide | 9.708 | 0.106 | 0.034 | 0.068 | no |
| ALDH1A1 | aldehyde dehydrogenase 1 family member A1 | 5.654 | 0.106 | 0.065 | 0.068 | no |
| NFATC3 | nuclear factor of activated T cells 3 | 4.073 | 0.106 | 0.040 | 0.107 | no |
| FCGR2A | Fc fragment of IgG receptor IIa | 5.863 | 0.105 | 0.030 | 0.093 | yes |
| IL17A | interleukin 17A | 4.611 | 0.105 | 0.047 | 0.096 | yes |
| DCTN1 | dynactin subunit 1 | 9.173 | 0.105 | 0.065 | 0.071 | no |
| NMNAT1 | nicotinamide nucleotide adenylyltransferase 1 | 6.067 | 0.105 | 0.046 | 0.082 | no |
| PPP1R9B | protein phosphatase 1 regulatory subunit 9B | 9.687 | 0.105 | 0.060 | 0.080 | no |
| PARP1 | poly(ADP-ribose) polymerase 1 | 5.520 | 0.105 | 0.057 | 0.069 | yes |
| PRKAB1 | protein kinase AMP-activated non-catalytic subunit beta 1 | 6.060 | 0.104 | 0.058 | 0.094 | yes |
| PIK3AP1 | phosphoinositide-3-kinase adaptor protein 1 | 8.423 | 0.104 | 0.045 | 0.073 | no |
| GZMH | granzyme H | 7.653 | 0.104 | 0.037 | 0.089 | no |
| TMSB10 | thymosin beta 10 | 8.168 | 0.104 | 0.060 | 0.079 | no |
| FOXO1 | forkhead box O1 | 7.754 | 0.104 | 0.066 | 0.084 | no |
| SERPINA9 | serpin family A member 9 | 6.221 | 0.103 | 0.040 | 0.078 | no |
| SCGB3A1 | secretoglobin family 3A member 1 | 6.869 | 0.103 | 0.027 | 0.082 | no |
| CLEC6A | C-type lectin domain containing 6A | 5.096 | 0.102 | 0.045 | 0.088 | no |
| FGF19 | fibroblast growth factor 19 | 10.939 | 0.102 | 0.054 | 0.079 | no |
| CNTNAP2 | contactin associated protein like 2 | 4.925 | 0.102 | 0.028 | 0.085 | no |
| MMP9 | matrix metallopeptidase 9 | 7.968 | 0.102 | 0.040 | 0.077 | yes |
| DPP7 | dipeptidyl peptidase 7 | 4.604 | 0.102 | 0.050 | 0.073 | no |
| SERPINE1 | serpin family E member 1 | 9.035 | 0.101 | 0.047 | 0.074 | yes |
| IL17C | interleukin 17C | 4.444 | 0.101 | 0.053 | 0.111 | no |
| SH2D1A | SH2 domain containing 1A | 5.445 | 0.101 | 0.043 | 0.105 | no |
| CCL7 | C-C motif chemokine ligand 7 | 4.991 | 0.101 | 0.047 | 0.108 | no |
| PAPPA | pappalysin 1 | 7.146 | 0.100 | 0.031 | 0.067 | no |
| IL1RN | interleukin 1 receptor antagonist | 7.579 | 0.100 | 0.035 | 0.078 | no |
| MMP1 | matrix metallopeptidase 1 | 13.678 | 0.100 | 0.039 | 0.073 | yes |
| CSF3 | colony stimulating factor 3 | 5.529 | 0.099 | 0.048 | 0.094 | no |
| CXADR | CXADR, Ig-like cell adhesion molecule | 5.149 | 0.099 | 0.036 | 0.080 | no |
| PECAM1 | platelet and endothelial cell adhesion molecule 1 | 8.815 | 0.099 | 0.061 | 0.069 | no |
| FXYD5 | FXYD domain containing ion transport regulator 5 | 5.422 | 0.099 | 0.056 | 0.077 | no |
| FOSB | FosB proto-oncogene, AP-1 transcription factor subunit | 3.597 | 0.098 | 0.043 | 0.165 | no |
| SAA4 | serum amyloid A4, constitutive | 6.213 | 0.098 | 0.036 | 0.067 | no |
| SOD1 | superoxide dismutase 1 | 5.216 | 0.098 | 0.058 | 0.090 | no |
| GPC5 | glypican 5 | 7.860 | 0.098 | 0.031 | 0.071 | no |
| TOP2B | DNA topoisomerase II beta | 6.934 | 0.097 | 0.052 | 0.065 | yes |
| UMOD | uromodulin | 4.479 | 0.097 | 0.038 | 0.062 | no |
| ARSB | arylsulfatase B | 5.293 | 0.097 | 0.054 | 0.077 | no |
| CPXM1 | carboxypeptidase X, M14 family member 1 | 8.143 | 0.097 | 0.047 | 0.079 | no |
| FETUB | fetuin B | 4.470 | 0.097 | 0.046 | 0.075 | no |
| CXCL11 | C-X-C motif chemokine ligand 11 | 11.406 | 0.097 | 0.045 | 0.073 | no |
| ARHGEF12 | Rho guanine nucleotide exchange factor 12 | 9.506 | 0.096 | 0.060 | 0.086 | no |
| PON2 | paraoxonase 2 | 5.412 | 0.096 | 0.032 | 0.076 | no |
| FCGR3B | Fc fragment of IgG receptor IIIb | 7.250 | 0.096 | 0.026 | 0.074 | yes |
| ITGAM | integrin subunit alpha M | 4.554 | 0.095 | 0.057 | 0.074 | no |
| GNLY | granulysin | 4.344 | 0.095 | 0.046 | 0.092 | no |
| PPP1R2 | protein phosphatase 1 regulatory inhibitor subunit 2 | 8.625 | 0.095 | 0.062 | 0.076 | no |
| FABP4 | fatty acid binding protein 4 | 8.483 | 0.095 | 0.034 | 0.072 | no |
| ARTN | artemin | 3.524 | 0.095 | 0.052 | 0.132 | no |
| PLIN1 | perilipin 1 | 6.248 | 0.094 | 0.039 | 0.077 | no |
| CD69 | CD69 molecule | 9.647 | 0.094 | 0.053 | 0.077 | no |
| CLMP | CXADR like membrane protein | 6.575 | 0.094 | 0.036 | 0.066 | no |
| FCRL6 | Fc receptor like 6 | 6.929 | 0.094 | 0.026 | 0.072 | no |
| IL17RB | interleukin 17 receptor B | 8.403 | 0.094 | 0.021 | 0.070 | no |
| DSG4 | desmoglein 4 | 6.960 | 0.094 | 0.027 | 0.084 | no |
| APLP1 | amyloid beta precursor like protein 1 | 10.589 | 0.094 | 0.045 | 0.053 | no |
| BST1 | bone marrow stromal cell antigen 1 | 4.835 | 0.094 | 0.044 | 0.068 | no |
| CSTB | cystatin B | 7.049 | 0.094 | 0.038 | 0.068 | no |
| CLEC7A | C-type lectin domain containing 7A | 6.379 | 0.094 | 0.023 | 0.090 | no |
| RRM2B | ribonucleotide reductase regulatory TP53 inducible subunit M2B | 5.343 | 0.093 | 0.053 | 0.070 | yes |
| TPSAB1 | tryptase alpha/beta 1 | 7.670 | 0.093 | 0.019 | 0.085 | no |
| KAZALD1 | Kazal type serine peptidase inhibitor domain 1 | 7.183 | 0.093 | 0.022 | 0.084 | no |
| ACP6 | acid phosphatase 6, lysophosphatidic | 7.520 | 0.093 | 0.028 | 0.070 | no |
| PRSS2 | serine protease 2 | 5.761 | 0.093 | 0.036 | 0.076 | no |
| FUCA1 | alpha-L-fucosidase 1 | 9.340 | 0.093 | 0.027 | 0.082 | no |
| IRF9 | interferon regulatory factor 9 | 5.800 | 0.093 | 0.051 | 0.065 | no |
| VIM | vimentin | 7.477 | 0.093 | 0.049 | 0.073 | no |
| AZU1 | azurocidin 1 | 5.350 | 0.093 | 0.042 | 0.076 | no |
| CD70 | CD70 molecule | 6.903 | 0.092 | 0.034 | 0.063 | no |
| CCL17 | C-C motif chemokine ligand 17 | 12.622 | 0.092 | 0.037 | 0.066 | no |
| TNFSF11 | TNF superfamily member 11 | 7.914 | 0.092 | 0.035 | 0.071 | yes |
| SUMF2 | sulfatase modifying factor 2 | 8.535 | 0.092 | 0.045 | 0.065 | no |
| ITGB1BP1 | integrin subunit beta 1 binding protein 1 | 4.247 | 0.092 | 0.066 | 0.083 | no |
| SLAMF1 | signaling lymphocytic activation molecule family member 1 | 4.746 | 0.092 | 0.034 | 0.070 | no |
| NOS3 | nitric oxide synthase 3 | 4.513 | 0.092 | 0.054 | 0.069 | yes |
| BLMH | bleomycin hydrolase | 5.619 | 0.091 | 0.053 | 0.067 | no |
| YES1 | YES proto-oncogene 1, Src family tyrosine kinase | 11.884 | 0.091 | 0.054 | 0.074 | yes |
| TFF2 | trefoil factor 2 | 7.598 | 0.090 | 0.027 | 0.076 | no |
| IL1RL1 | interleukin 1 receptor like 1 | 7.449 | 0.090 | 0.029 | 0.069 | no |
| PSIP1 | PC4 and SFRS1 interacting protein 1 | 5.518 | 0.090 | 0.040 | 0.077 | no |
| DRAXIN | dorsal inhibitory axon guidance protein | 6.345 | 0.090 | 0.028 | 0.063 | no |
| FAP | fibroblast activation protein alpha | 4.355 | 0.090 | 0.048 | 0.070 | no |
| NADK | NAD kinase | 6.478 | 0.089 | 0.039 | 0.072 | no |
| DDAH1 | dimethylarginine dimethylaminohydrolase 1 | 5.142 | 0.089 | 0.053 | 0.107 | no |
| OMD | osteomodulin | 8.272 | 0.089 | 0.027 | 0.071 | no |
| SIT1 | signaling threshold regulating transmembrane adaptor 1 | 5.857 | 0.089 | 0.039 | 0.067 | no |
| NAAA | N-acylethanolamine acid amidase | 6.999 | 0.089 | 0.030 | 0.076 | no |
| ARG1 | arginase 1 | 5.239 | 0.089 | 0.051 | 0.072 | no |
| APEX1 | apurinic/apyrimidinic endodeoxyribonuclease 1 | 4.497 | 0.089 | 0.053 | 0.063 | yes |
| SLAMF8 | SLAM family member 8 | 5.517 | 0.089 | 0.030 | 0.073 | no |
| CSF2RA | colony stimulating factor 2 receptor alpha subunit | 9.758 | 0.088 | 0.013 | 0.068 | yes |
| LAMP3 | lysosomal associated membrane protein 3 | 7.468 | 0.088 | 0.028 | 0.063 | no |
| PPIB | peptidylprolyl isomerase B | 8.759 | 0.088 | 0.060 | 0.079 | no |
| CADM3 | cell adhesion molecule 3 | 6.197 | 0.088 | 0.028 | 0.068 | no |
| MVK | mevalonate kinase | 7.622 | 0.087 | 0.051 | 0.069 | no |
| PRTN3 | proteinase 3 | 6.822 | 0.087 | 0.029 | 0.059 | no |
| ASAH2 | N-acylsphingosine amidohydrolase 2 | 7.365 | 0.087 | 0.022 | 0.094 | no |
| IL10RA | interleukin 10 receptor subunit alpha | 3.977 | 0.087 | 0.044 | 0.134 | no |
| TNR | tenascin R | 7.971 | 0.086 | 0.022 | 0.064 | no |
| IL2RB | interleukin 2 receptor subunit beta | 4.504 | 0.086 | 0.045 | 0.113 | yes |
| HEXIM1 | hexamethylene bisacetamide inducible 1 | 9.976 | 0.086 | 0.050 | 0.066 | no |
| FCRL3 | Fc receptor like 3 | 5.054 | 0.086 | 0.025 | 0.071 | no |
| CEACAM8 | carcinoembryonic antigen related cell adhesion molecule 8 | 6.898 | 0.086 | 0.038 | 0.056 | no |
| CCL18 | C-C motif chemokine ligand 18 | 9.035 | 0.086 | 0.026 | 0.071 | no |
| CLUL1 | clusterin like 1 | 7.477 | 0.086 | 0.025 | 0.062 | no |
| GLRX | glutaredoxin | 4.508 | 0.086 | 0.052 | 0.060 | no |
| IL12B | interleukin 12B | 10.833 | 0.086 | 0.015 | 0.048 | yes |
| ANXA4 | annexin A4 | 5.258 | 0.085 | 0.055 | 0.066 | no |
| NPPC | natriuretic peptide C | 6.672 | 0.085 | 0.030 | 0.068 | no |
| PXN | paxillin | 5.858 | 0.085 | 0.034 | 0.063 | no |
| ENO2 | enolase 2 | 9.145 | 0.085 | 0.056 | 0.068 | no |
| CXCL6 | C-X-C motif chemokine ligand 6 | 12.414 | 0.085 | 0.040 | 0.061 | no |
| IL20RA | interleukin 20 receptor subunit alpha | 4.067 | 0.085 | 0.045 | 0.088 | no |
| SNAP29 | synaptosome associated protein 29 | 10.387 | 0.084 | 0.051 | 0.075 | no |
| PLA2G7 | phospholipase A2 group VII | 4.616 | 0.084 | 0.046 | 0.061 | no |
| BANK1 | B cell scaffold protein with ankyrin repeats 1 | 11.671 | 0.084 | 0.055 | 0.067 | no |
| CD300LF | CD300 molecule like family member f | 8.779 | 0.084 | 0.020 | 0.062 | no |
| WFIKKN1 | WAP, follistatin/kazal, immunoglobulin, kunitz and netrin domain containing 1 | 6.253 | 0.084 | 0.020 | 0.060 | no |
| RTN4R | reticulon 4 receptor | 5.232 | 0.083 | 0.033 | 0.059 | no |
| GALNT3 | polypeptide N-acetylgalactosaminyltransferase 3 | 4.905 | 0.083 | 0.047 | 0.065 | no |
| CPA1 | carboxypeptidase A1 | 9.123 | 0.083 | 0.028 | 0.073 | no |
| FUT3/5 | fucosyltransferase 3/5 | 6.943 | 0.083 | 0.027 | 0.062 | no |
| ROR1 | receptor tyrosine kinase like orphan receptor 1 | 5.828 | 0.083 | 0.034 | 0.057 | no |
| FGF23 | fibroblast growth factor 23 | 5.871 | 0.083 | 0.036 | 0.062 | no |
| CPB1 | carboxypeptidase B1 | 8.922 | 0.083 | 0.028 | 0.067 | no |
| MMP3 | matrix metallopeptidase 3 | 10.560 | 0.083 | 0.023 | 0.060 | yes |
| MSTN | myostatin | 7.940 | 0.083 | 0.025 | 0.058 | no |
| LIF | LIF, interleukin 6 family cytokine | 3.356 | 0.083 | 0.048 | 0.077 | no |
| CNDP1 | carnosine dipeptidase 1 | 6.104 | 0.083 | 0.038 | 0.061 | no |
| HSD11B1 | hydroxysteroid 11-beta dehydrogenase 1 | 6.085 | 0.083 | 0.026 | 0.065 | no |
| XG | Xg blood group | 8.053 | 0.082 | 0.024 | 0.054 | no |
| APBB1IP | amyloid beta precursor protein binding family B member 1 interacting protein | 5.124 | 0.082 | 0.037 | 0.074 | no |
| IL17RA | interleukin 17 receptor A | 7.392 | 0.082 | 0.032 | 0.066 | no |
| TIGAR | TP53 induced glycolysis regulatory phosphatase | 4.746 | 0.082 | 0.043 | 0.071 | no |
| PILRB | paired immunoglobin-like type 2 receptor beta | 8.915 | 0.081 | 0.025 | 0.061 | no |
| TIMD4 | T cell immunoglobulin and mucin domain containing 4 | 6.775 | 0.081 | 0.027 | 0.063 | no |
| CALR | calreticulin | 3.721 | 0.081 | 0.050 | 0.050 | no |
| DKK4 | dickkopf WNT signaling pathway inhibitor 4 | 6.698 | 0.081 | 0.036 | 0.059 | no |
| PON3 | paraoxonase 3 | 10.030 | 0.081 | 0.024 | 0.057 | no |
| NUCB2 | nucleobindin 2 | 8.414 | 0.081 | 0.042 | 0.057 | no |
| VAMP5 | vesicle associated membrane protein 5 | 4.517 | 0.080 | 0.045 | 0.075 | no |
| SKAP1 | src kinase associated phosphoprotein 1 | 8.788 | 0.080 | 0.030 | 0.058 | no |
| IGFBP2 | insulin like growth factor binding protein 2 | 11.154 | 0.080 | 0.022 | 0.055 | no |
| TNFRSF10C | TNF receptor superfamily member 10c | 10.003 | 0.080 | 0.019 | 0.054 | no |
| THBS4 | thrombospondin 4 | 8.130 | 0.079 | 0.028 | 0.064 | no |
| MGMT | O-6-methylguanine-DNA methyltransferase | 9.909 | 0.079 | 0.046 | 0.081 | no |
| TRIM21 | tripartite motif containing 21 | 7.771 | 0.079 | 0.049 | 0.065 | no |
| MILR1 | mast cell immunoglobulin like receptor 1 | 6.603 | 0.079 | 0.022 | 0.060 | no |
| CLSPN | claspin | 4.107 | 0.079 | 0.049 | 0.107 | no |
| SERPINB6 | serpin family B member 6 | 8.226 | 0.079 | 0.051 | 0.056 | no |
| HCLS1 | hematopoietic cell-specific Lyn substrate 1 | 10.088 | 0.079 | 0.048 | 0.065 | no |
| CXCL9 | C-X-C motif chemokine ligand 9 | 10.218 | 0.079 | 0.033 | 0.079 | no |
| FCRL1 | Fc receptor like 1 | 8.281 | 0.079 | 0.022 | 0.056 | no |
| REG3A | regenerating family member 3 alpha | 3.534 | 0.079 | 0.048 | 0.063 | no |
| TGFBR3 | transforming growth factor beta receptor 3 | 5.533 | 0.078 | 0.039 | 0.066 | no |
| MPO | myeloperoxidase | 6.076 | 0.078 | 0.032 | 0.075 | no |
| LILRB1 | leukocyte immunoglobulin like receptor B1 | 4.301 | 0.078 | 0.038 | 0.055 | no |
| AGRP | agouti related neuropeptide | 7.156 | 0.078 | 0.039 | 0.054 | no |
| NTF4 | neurotrophin 4 | 5.066 | 0.078 | 0.041 | 0.084 | no |
| HPGDS | hematopoietic prostaglandin D synthase | 5.451 | 0.078 | 0.026 | 0.057 | no |
| DGKZ | diacylglycerol kinase zeta | 3.479 | 0.078 | 0.050 | 0.098 | no |
| CA2 | carbonic anhydrase 2 | 9.669 | 0.077 | 0.041 | 0.056 | yes |
| FCER2 | Fc fragment of IgE receptor II | 8.712 | 0.077 | 0.020 | 0.061 | no |
| SIRPA | signal regulatory protein alpha | 6.875 | 0.077 | 0.027 | 0.054 | no |
| FAM19A5 | family with sequence similarity 19 member A5, C-C motif chemokine like | 6.244 | 0.077 | 0.035 | 0.052 | no |
| TP53 | tumor protein p53 | 6.470 | 0.077 | 0.051 | 0.056 | no |
| LRRN1 | leucine rich repeat neuronal 1 | 7.456 | 0.077 | 0.024 | 0.059 | no |
| CD207 | CD207 molecule | 7.092 | 0.077 | 0.025 | 0.053 | no |
| CDCP1 | CUB domain containing protein 1 | 6.120 | 0.076 | 0.029 | 0.065 | no |
| PRCP | prolylcarboxypeptidase | 5.087 | 0.076 | 0.048 | 0.074 | no |
| IL10 | interleukin 10 | 6.635 | 0.076 | 0.036 | 0.081 | no |
| FCRL5 | Fc receptor like 5 | 7.870 | 0.076 | 0.022 | 0.059 | no |
| BIRC2 | baculoviral IAP repeat containing 2 | 4.074 | 0.076 | 0.042 | 0.052 | no |
| DLK1 | delta like non-canonical Notch ligand 1 | 9.084 | 0.076 | 0.025 | 0.057 | no |
| ANGPT2 | angiopoietin 2 | 5.819 | 0.076 | 0.033 | 0.059 | no |
| LCN2 | lipocalin 2 | 5.144 | 0.076 | 0.033 | 0.058 | no |
| CYR61 | cysteine rich angiogenic inducer 61 | 8.883 | 0.075 | 0.042 | 0.050 | no |
| DPP6 | dipeptidyl peptidase like 6 | 6.164 | 0.075 | 0.024 | 0.057 | no |
| IL4R | interleukin 4 receptor | 5.530 | 0.075 | 0.046 | 0.090 | yes |
| ACE2 | angiotensin I converting enzyme 2 | 7.453 | 0.075 | 0.023 | 0.058 | yes |
| CCL25 | C-C motif chemokine ligand 25 | 9.179 | 0.075 | 0.022 | 0.054 | no |
| CDSN | corneodesmosin | 6.300 | 0.075 | 0.034 | 0.059 | no |
| EGLN1 | egl-9 family hypoxia inducible factor 1 | 4.546 | 0.075 | 0.038 | 0.057 | no |
| ADGRG1 | adhesion G protein-coupled receptor G1 | 4.915 | 0.074 | 0.039 | 0.078 | no |
| BGN | biglycan | 5.589 | 0.074 | 0.038 | 0.065 | no |
| CCL28 | C-C motif chemokine ligand 28 | 5.367 | 0.074 | 0.028 | 0.056 | no |
| SERPINB8 | serpin family B member 8 | 6.338 | 0.074 | 0.033 | 0.059 | no |
| TLR3 | toll like receptor 3 | 9.299 | 0.074 | 0.015 | 0.067 | no |
| ANGPTL4 | angiopoietin like 4 | 7.828 | 0.074 | 0.033 | 0.056 | no |
| LGALS4 | galectin 4 | 6.854 | 0.073 | 0.040 | 0.056 | no |
| PADI2 | peptidyl arginine deiminase 2 | 4.218 | 0.073 | 0.039 | 0.120 | no |
| PTX3 | pentraxin 3 | 7.470 | 0.073 | 0.030 | 0.059 | no |
| PTK7 | protein tyrosine kinase 7 (inactive) | 4.993 | 0.073 | 0.043 | 0.079 | no |
| NUDT5 | nudix hydrolase 5 | 7.729 | 0.073 | 0.050 | 0.063 | no |
| NCF2 | neutrophil cytosolic factor 2 | 7.051 | 0.073 | 0.033 | 0.055 | no |
| GIF | gastric intrinsic factor | 10.478 | 0.072 | 0.023 | 0.053 | no |
| IL2RA | interleukin 2 receptor subunit alpha | 6.530 | 0.072 | 0.029 | 0.053 | yes |
| CRTAM | cytotoxic and regulatory T cell molecule | 8.108 | 0.072 | 0.026 | 0.059 | no |
| SORCS2 | sortilin related VPS10 domain containing receptor 2 | 5.192 | 0.072 | 0.031 | 0.066 | no |
| NCR1 | natural cytotoxicity triggering receptor 1 | 5.566 | 0.072 | 0.029 | 0.054 | no |
| MIF | macrophage migration inhibitory factor | 11.662 | 0.072 | 0.052 | 0.063 | no |
| CST5 | cystatin D | 8.583 | 0.072 | 0.021 | 0.052 | no |
| MFAP5 | microfibril associated protein 5 | 4.630 | 0.072 | 0.042 | 0.052 | no |
| LILRB4 | leukocyte immunoglobulin like receptor B4 | 6.985 | 0.072 | 0.020 | 0.057 | no |
| MMP12 | matrix metallopeptidase 12 | 9.558 | 0.072 | 0.022 | 0.069 | yes |
| COL1A1 | collagen type I alpha 1 chain | 6.215 | 0.072 | 0.031 | 0.053 | yes |
| CSNK1D | casein kinase 1 delta | 4.783 | 0.071 | 0.038 | 0.117 | no |
| ZBTB17 | zinc finger and BTB domain containing 17 | 5.669 | 0.071 | 0.039 | 0.056 | no |
| SPINK1 | serine peptidase inhibitor, Kazal type 1 | 7.661 | 0.071 | 0.027 | 0.050 | no |
| KLRD1 | killer cell lectin like receptor D1 | 9.959 | 0.071 | 0.015 | 0.052 | no |
| REN | renin | 11.546 | 0.071 | 0.022 | 0.053 | yes |
| DPP10 | dipeptidyl peptidase like 10 | 4.822 | 0.071 | 0.029 | 0.106 | no |
| CES2 | carboxylesterase 2 | 4.814 | 0.071 | 0.035 | 0.094 | no |
| ANXA1 | annexin A1 | 7.242 | 0.071 | 0.043 | 0.050 | yes |
| FCN2 | ficolin 2 | 8.934 | 0.071 | 0.021 | 0.053 | no |
| GCNT1 | glucosaminyl (N-acetyl) transferase 1, core 2 | 5.177 | 0.071 | 0.027 | 0.060 | no |
| FRZB | frizzled related protein | 6.443 | 0.071 | 0.024 | 0.052 | no |
| LGALS7 | galectin 7 | 6.847 | 0.071 | 0.032 | 0.053 | no |
| CA1 | carbonic anhydrase 1 | 8.970 | 0.070 | 0.038 | 0.053 | yes |
| CD6 | CD6 molecule | 8.692 | 0.070 | 0.030 | 0.048 | no |
| VWC2 | von Willebrand factor C domain containing 2 | 7.643 | 0.070 | 0.023 | 0.056 | no |
| KLK10 | kallikrein related peptidase 10 | 6.397 | 0.070 | 0.031 | 0.047 | no |
| NBN | nibrin | 5.461 | 0.070 | 0.036 | 0.066 | no |
| SEZ6L2 | seizure related 6 homolog like 2 | 3.464 | 0.070 | 0.035 | 0.051 | no |
| SELP | selectin P | 14.109 | 0.069 | 0.042 | 0.051 | no |
| VSIG2 | V-set and immunoglobulin domain containing 2 | 7.167 | 0.069 | 0.033 | 0.052 | no |
| CD300LG | CD300 molecule like family member g | 8.515 | 0.069 | 0.022 | 0.047 | no |
| KLK6 | kallikrein related peptidase 6 | 5.680 | 0.069 | 0.038 | 0.055 | no |
| INHBC | inhibin beta C subunit | 7.145 | 0.069 | 0.031 | 0.052 | no |
| ADAM23 | ADAM metallopeptidase domain 23 | 8.011 | 0.069 | 0.021 | 0.051 | no |
| SLAMF7 | SLAM family member 7 | 7.197 | 0.069 | 0.028 | 0.055 | yes |
| MASP1 | mannan binding lectin serine peptidase 1 | 5.790 | 0.069 | 0.021 | 0.047 | no |
| CRELD2 | cysteine rich with EGF like domains 2 | 7.505 | 0.069 | 0.039 | 0.047 | no |
| ACP5 | acid phosphatase 5, tartrate resistant | 6.513 | 0.069 | 0.028 | 0.049 | no |
| DDC | dopa decarboxylase | 9.526 | 0.069 | 0.029 | 0.051 | yes |
| IGFBPL1 | insulin like growth factor binding protein like 1 | 6.149 | 0.069 | 0.025 | 0.053 | no |
| LILRB2 | leukocyte immunoglobulin like receptor B2 | 6.578 | 0.069 | 0.025 | 0.057 | no |
| FCRL2 | Fc receptor like 2 | 8.213 | 0.069 | 0.017 | 0.055 | no |
| OPTC | opticin | 7.454 | 0.069 | 0.018 | 0.056 | no |
| NRTN | neurturin | 3.745 | 0.069 | 0.047 | 0.129 | no |
| TMPRSS5 | transmembrane serine protease 5 | 7.192 | 0.069 | 0.019 | 0.053 | no |
| ADAMTS15 | ADAM metallopeptidase with thrombospondin type 1 motif 15 | 7.626 | 0.068 | 0.034 | 0.059 | no |
| KLK13 | kallikrein related peptidase 13 | 7.766 | 0.068 | 0.027 | 0.051 | no |
| MYOC | myocilin | 9.051 | 0.068 | 0.026 | 0.049 | no |
| CR2 | complement C3d receptor 2 | 8.715 | 0.068 | 0.020 | 0.053 | no |
| IL33 | interleukin 33 | 3.790 | 0.068 | 0.046 | 0.057 | no |
| TNFRSF10A | TNF receptor superfamily member 10a | 5.576 | 0.068 | 0.029 | 0.045 | no |
| GDNF | glial cell derived neurotrophic factor | 5.555 | 0.068 | 0.035 | 0.056 | no |
| ARSA | arylsulfatase A | 7.398 | 0.068 | 0.032 | 0.058 | no |
| CST6 | cystatin E/M | 8.680 | 0.068 | 0.020 | 0.052 | no |
| CXCL5 | C-X-C motif chemokine ligand 5 | 15.439 | 0.068 | 0.032 | 0.053 | no |
| ANGPTL7 | angiopoietin like 7 | 4.441 | 0.067 | 0.040 | 0.047 | no |
| IL12RB1 | interleukin 12 receptor subunit beta 1 | 5.517 | 0.067 | 0.024 | 0.051 | no |
| CTSF | cathepsin F | 7.095 | 0.067 | 0.031 | 0.050 | no |
| MFGE8 | milk fat globule-EGF factor 8 protein | 7.410 | 0.067 | 0.032 | 0.055 | no |
| MEGF9 | multiple EGF like domains 9 | 5.273 | 0.067 | 0.042 | 0.048 | no |
| TFRC | transferrin receptor | 8.863 | 0.067 | 0.030 | 0.050 | no |
| ITGB6 | integrin subunit beta 6 | 6.258 | 0.067 | 0.029 | 0.046 | no |
| SLITRK2 | SLIT and NTRK like family member 2 | 7.977 | 0.067 | 0.021 | 0.044 | no |
| KYAT1 | kynurenine aminotransferase 1 | 11.446 | 0.067 | 0.039 | 0.046 | no |
| IL2 | interleukin 2 | 3.502 | 0.066 | 0.047 | 0.052 | no |
| FABP2 | fatty acid binding protein 2 | 12.360 | 0.066 | 0.032 | 0.052 | no |
| CCL3 | C-C motif chemokine ligand 3 | 7.798 | 0.066 | 0.030 | 0.091 | no |
| CNTN2 | contactin 2 | 7.738 | 0.066 | 0.022 | 0.048 | no |
| AREG | amphiregulin | 5.624 | 0.066 | 0.034 | 0.065 | no |
| CLEC1A | C-type lectin domain family 1 member A | 5.847 | 0.066 | 0.030 | 0.049 | no |
| ST6GAL1 | ST6 beta-galactoside alpha-2,6-sialyltransferase 1 | 6.139 | 0.065 | 0.038 | 0.047 | no |
| SOST | sclerostin | 8.187 | 0.065 | 0.028 | 0.045 | no |
| SMPD1 | sphingomyelin phosphodiesterase 1 | 8.772 | 0.065 | 0.021 | 0.050 | no |
| CCL4 | C-C motif chemokine ligand 4 | 8.913 | 0.065 | 0.028 | 0.062 | no |
| PDCD1 | programmed cell death 1 | 3.621 | 0.065 | 0.036 | 0.056 | yes |
| IL12B | interleukin 12B | 8.887 | 0.065 | 0.020 | 0.057 | yes |
| XCL1 | X-C motif chemokine ligand 1 | 8.859 | 0.065 | 0.018 | 0.069 | no |
| MCFD2 | multiple coagulation factor deficiency 2 | 6.908 | 0.065 | 0.036 | 0.050 | no |
| FGF5 | fibroblast growth factor 5 | 4.096 | 0.065 | 0.034 | 0.130 | no |
| RASSF2 | Ras association domain family member 2 | 4.939 | 0.065 | 0.032 | 0.053 | no |
| C1QTNF1 | C1q and TNF related 1 | 6.527 | 0.065 | 0.038 | 0.050 | no |
| LGALS3 | galectin 3 | 6.380 | 0.065 | 0.029 | 0.048 | no |
| ENTPD2 | ectonucleoside triphosphate diphosphohydrolase 2 | 4.226 | 0.065 | 0.035 | 0.054 | no |
| ITGA11 | integrin subunit alpha 11 | 6.821 | 0.064 | 0.021 | 0.050 | no |
| CD109 | CD109 molecule | 7.873 | 0.064 | 0.033 | 0.042 | no |
| CLEC4C | C-type lectin domain family 4 member C | 7.075 | 0.064 | 0.019 | 0.055 | no |
| CFC1 | cripto, FRL-1, cryptic family 1 | 4.049 | 0.063 | 0.034 | 0.062 | no |
| CCL19 | C-C motif chemokine ligand 19 | 11.681 | 0.063 | 0.023 | 0.063 | no |
| GKN1 | gastrokine 1 | 3.337 | 0.063 | 0.036 | 0.048 | no |
| CCL8 | C-C motif chemokine ligand 8 | 11.730 | 0.063 | 0.025 | 0.050 | no |
| BCAN | brevican | 9.556 | 0.063 | 0.017 | 0.042 | no |
| COCH | cochlin | 7.462 | 0.063 | 0.030 | 0.065 | no |
| CTSO | cathepsin O | 5.761 | 0.063 | 0.030 | 0.050 | no |
| BCL2L11 | BCL2 like 11 | 6.028 | 0.063 | 0.030 | 0.052 | no |
| TNFRSF6B | TNF receptor superfamily member 6b | 8.215 | 0.062 | 0.024 | 0.053 | no |
| CHL1 | cell adhesion molecule L1 like | 6.582 | 0.062 | 0.027 | 0.044 | no |
| CTSV | cathepsin V | 8.109 | 0.062 | 0.025 | 0.050 | no |
| ITGB7 | integrin subunit beta 7 | 4.439 | 0.062 | 0.028 | 0.049 | yes |
| PLXNB2 | plexin B2 | 4.637 | 0.062 | 0.037 | 0.052 | no |
| RET | ret proto-oncogene | 8.865 | 0.062 | 0.019 | 0.045 | yes |
| SIRPB1 | signal regulatory protein beta 1 | 7.072 | 0.062 | 0.025 | 0.055 | no |
| SPINT2 | serine peptidase inhibitor, Kunitz type 2 | 5.864 | 0.062 | 0.041 | 0.054 | no |
| RSPO1 | R-spondin 1 | 6.830 | 0.062 | 0.021 | 0.045 | no |
| ADAM22 | ADAM metallopeptidase domain 22 | 7.421 | 0.062 | 0.021 | 0.047 | no |
| CLEC10A | C-type lectin domain containing 10A | 7.536 | 0.062 | 0.020 | 0.047 | no |
| PTPRS | protein tyrosine phosphatase, receptor type S | 4.050 | 0.062 | 0.037 | 0.052 | no |
| SPON1 | spondin 1 | 5.048 | 0.061 | 0.032 | 0.044 | no |
| PDP1 | pyruvate dehyrogenase phosphatase catalytic subunit 1 | 3.815 | 0.061 | 0.044 | 0.047 | no |
| ADGRE2 | adhesion G protein-coupled receptor E2 | 6.602 | 0.061 | 0.029 | 0.049 | no |
| MB | myoglobin | 10.863 | 0.061 | 0.024 | 0.047 | no |
| CD160 | CD160 molecule | 9.027 | 0.061 | 0.017 | 0.046 | no |
| LRP11 | LDL receptor related protein 11 | 7.689 | 0.061 | 0.026 | 0.039 | no |
| IL15RA | interleukin 15 receptor subunit alpha | 4.789 | 0.061 | 0.032 | 0.045 | no |
| MOG | myelin oligodendrocyte glycoprotein | 7.050 | 0.061 | 0.025 | 0.046 | no |
| GDF2 | growth differentiation factor 2 | 11.449 | 0.061 | 0.016 | 0.045 | no |
| ADGRG2 | adhesion G protein-coupled receptor G2 | 6.749 | 0.061 | 0.029 | 0.041 | no |
| COL4A1 | collagen type IV alpha 1 chain | 9.764 | 0.060 | 0.021 | 0.047 | no |
| CDH2 | cadherin 2 | 7.303 | 0.060 | 0.028 | 0.044 | no |
| CXCL12 | C-X-C motif chemokine ligand 12 | 4.387 | 0.060 | 0.034 | 0.045 | no |
| DKK3 | dickkopf WNT signaling pathway inhibitor 3 | 8.943 | 0.060 | 0.022 | 0.044 | no |
| LYVE1 | lymphatic vessel endothelial hyaluronan receptor 1 | 8.231 | 0.060 | 0.020 | 0.046 | no |
| SIRT5 | sirtuin 5 | 3.825 | 0.060 | 0.044 | 0.051 | yes |
| CD38 | CD38 molecule | 7.831 | 0.060 | 0.022 | 0.046 | no |
| TNFRSF4 | TNF receptor superfamily member 4 | 7.590 | 0.060 | 0.023 | 0.046 | no |
| ULBP2 | UL16 binding protein 2 | 6.906 | 0.060 | 0.018 | 0.041 | no |
| CDHR5 | cadherin related family member 5 | 7.327 | 0.060 | 0.019 | 0.050 | no |
| NOV | nephroblastoma overexpressed | 6.853 | 0.060 | 0.029 | 0.044 | no |
| ENTPD5 | ectonucleoside triphosphate diphosphohydrolase 5 | 6.054 | 0.060 | 0.030 | 0.045 | no |
| KLK14 | kallikrein related peptidase 14 | 10.760 | 0.060 | 0.019 | 0.042 | no |
| IL18 | interleukin 18 | 11.245 | 0.060 | 0.017 | 0.044 | no |
| CCL15 | C-C motif chemokine ligand 15 | 9.823 | 0.060 | 0.026 | 0.049 | no |
| ST3GAL1 | ST3 beta-galactoside alpha-2,3-sialyltransferase 1 | 7.576 | 0.059 | 0.021 | 0.056 | no |
| SPARCL1 | SPARC like 1 | 5.571 | 0.059 | 0.030 | 0.044 | no |
| TREML2 | triggering receptor expressed on myeloid cells like 2 | 8.869 | 0.059 | 0.028 | 0.041 | no |
| CLEC11A | C-type lectin domain containing 11A | 8.633 | 0.059 | 0.022 | 0.046 | no |
| NID1 | nidogen 1 | 7.037 | 0.059 | 0.031 | 0.046 | no |
| PILRA | paired immunoglobin like type 2 receptor alpha | 7.478 | 0.059 | 0.025 | 0.047 | no |
| ANGPTL1 | angiopoietin like 1 | 6.260 | 0.059 | 0.026 | 0.043 | no |
| PAM | peptidylglycine alpha-amidating monooxygenase | 4.950 | 0.059 | 0.035 | 0.048 | no |
| PLXDC1 | plexin domain containing 1 | 3.972 | 0.059 | 0.037 | 0.049 | no |
| RETN | resistin | 8.934 | 0.059 | 0.024 | 0.051 | no |
| CD209 | CD209 molecule | 9.984 | 0.059 | 0.016 | 0.046 | no |
| TNFRSF11B | TNF receptor superfamily member 11b | 6.822 | 0.059 | 0.026 | 0.047 | no |
| GALNT10 | polypeptide N-acetylgalactosaminyltransferase 10 | 4.643 | 0.059 | 0.032 | 0.041 | no |
| HS3ST3B1 | heparan sulfate-glucosamine 3-sulfotransferase 3B1 | 6.170 | 0.059 | 0.029 | 0.043 | no |
| IL1RL2 | interleukin 1 receptor like 2 | 8.122 | 0.058 | 0.020 | 0.046 | no |
| KIT | KIT proto-oncogene receptor tyrosine kinase | 7.553 | 0.058 | 0.025 | 0.043 | yes |
| VASN | vasorin | 5.673 | 0.058 | 0.029 | 0.044 | no |
| BAMBI | BMP and activin membrane bound inhibitor | 4.532 | 0.058 | 0.027 | 0.051 | no |
| NINJ1 | ninjurin 1 | 5.117 | 0.058 | 0.027 | 0.072 | no |
| CD83 | CD83 molecule | 6.423 | 0.058 | 0.025 | 0.046 | no |
| QPCT | glutaminyl-peptide cyclotransferase | 5.682 | 0.058 | 0.033 | 0.057 | no |
| NPTXR | neuronal pentraxin receptor | 7.232 | 0.058 | 0.023 | 0.053 | no |
| CCDC80 | coiled-coil domain containing 80 | 9.535 | 0.058 | 0.025 | 0.040 | no |
| TNXB | tenascin XB | 4.399 | 0.058 | 0.035 | 0.044 | no |
| XPNPEP2 | X-prolyl aminopeptidase 2 | 12.826 | 0.058 | 0.008 | 0.050 | no |
| STC1 | stanniocalcin 1 | 9.093 | 0.058 | 0.025 | 0.040 | no |
| LILRA5 | leukocyte immunoglobulin like receptor A5 | 8.121 | 0.058 | 0.021 | 0.041 | no |
| GDF15 | growth differentiation factor 15 | 8.373 | 0.057 | 0.023 | 0.039 | no |
| IGLC2 | NA | 9.193 | 0.057 | 0.019 | 0.042 | no |
| CXCL17 | C-X-C motif chemokine ligand 17 | 8.189 | 0.057 | 0.021 | 0.044 | no |
| CTSZ | cathepsin Z | 8.291 | 0.057 | 0.023 | 0.042 | no |
| S100P | S100 calcium binding protein P | 4.201 | 0.057 | 0.032 | 0.053 | no |
| ICAM2 | intercellular adhesion molecule 2 | 8.404 | 0.057 | 0.025 | 0.040 | no |
| LY75 | lymphocyte antigen 75 | 5.962 | 0.057 | 0.023 | 0.041 | no |
| CXCL10 | C-X-C motif chemokine ligand 10 | 12.552 | 0.057 | 0.027 | 0.065 | no |
| DSC2 | desmocollin 2 | 7.818 | 0.057 | 0.023 | 0.040 | no |
| CD84 | CD84 molecule | 10.276 | 0.057 | 0.030 | 0.040 | no |
| AOC3 | amine oxidase, copper containing 3 | 6.868 | 0.057 | 0.023 | 0.039 | yes |
| PDCD1LG2 | programmed cell death 1 ligand 2 | 6.599 | 0.057 | 0.021 | 0.042 | no |
| RSPO3 | R-spondin 3 | 7.834 | 0.056 | 0.022 | 0.061 | no |
| CLEC4G | C-type lectin domain family 4 member G | 6.391 | 0.056 | 0.024 | 0.049 | no |
| TCN2 | transcobalamin 2 | 6.416 | 0.056 | 0.024 | 0.046 | no |
| CLSTN3 | calsyntenin 3 | 4.429 | 0.056 | 0.032 | 0.049 | no |
| HAVCR2 | hepatitis A virus cellular receptor 2 | 6.774 | 0.056 | 0.025 | 0.048 | no |
| MMP10 | matrix metallopeptidase 10 | 11.512 | 0.056 | 0.023 | 0.048 | yes |
| FAM3B | family with sequence similarity 3 member B | 7.999 | 0.056 | 0.022 | 0.043 | no |
| PCDH17 | protocadherin 17 | 8.672 | 0.056 | 0.021 | 0.041 | no |
| KYNU | kynureninase | 10.775 | 0.056 | 0.023 | 0.043 | no |
| REG4 | regenerating family member 4 | 12.146 | 0.056 | 0.020 | 0.039 | no |
| IGFBP6 | insulin like growth factor binding protein 6 | 7.388 | 0.056 | 0.025 | 0.040 | no |
| GZMA | granzyme A | 7.953 | 0.056 | 0.021 | 0.044 | no |
| NCAM1 | neural cell adhesion molecule 1 | 6.610 | 0.055 | 0.026 | 0.041 | no |
| IGFBP3 | insulin like growth factor binding protein 3 | 8.038 | 0.055 | 0.020 | 0.037 | no |
| EFEMP1 | EGF containing fibulin extracellular matrix protein 1 | 7.113 | 0.055 | 0.026 | 0.044 | no |
| CNTN5 | contactin 5 | 10.480 | 0.055 | 0.012 | 0.040 | no |
| LRIG1 | leucine rich repeats and immunoglobulin like domains 1 | 7.198 | 0.055 | 0.023 | 0.041 | no |
| OLR1 | oxidized low density lipoprotein receptor 1 | 9.578 | 0.055 | 0.026 | 0.039 | no |
| NOMO1 | NODAL modulator 1 | 8.468 | 0.055 | 0.027 | 0.040 | no |
| VCAN | versican | 7.765 | 0.055 | 0.020 | 0.039 | no |
| HAVCR1 | hepatitis A virus cellular receptor 1 | 11.888 | 0.055 | 0.017 | 0.047 | no |
| CLSTN2 | calsyntenin 2 | 7.800 | 0.054 | 0.023 | 0.050 | no |
| SLITRK6 | SLIT and NTRK like family member 6 | 4.126 | 0.054 | 0.036 | 0.043 | no |
| SEZ6L | seizure related 6 homolog like | 7.487 | 0.054 | 0.017 | 0.038 | no |
| IL16 | interleukin 16 | 9.844 | 0.054 | 0.021 | 0.045 | no |
| ICAM3 | intercellular adhesion molecule 3 | 6.231 | 0.054 | 0.030 | 0.039 | no |
| CTRC | chymotrypsin C | 14.368 | 0.054 | 0.017 | 0.040 | no |
| PCOLCE | procollagen C-endopeptidase enhancer | 8.954 | 0.053 | 0.022 | 0.037 | no |
| COL18A1 | collagen type XVIII alpha 1 chain | 6.222 | 0.053 | 0.028 | 0.037 | no |
| TFF3 | trefoil factor 3 | 8.005 | 0.053 | 0.022 | 0.039 | no |
| IFNLR1 | interferon lambda receptor 1 | 5.724 | 0.053 | 0.023 | 0.069 | no |
| PDGFRB | platelet derived growth factor receptor beta | 9.053 | 0.053 | 0.016 | 0.037 | yes |
| LAG3 | lymphocyte activating 3 | 6.378 | 0.053 | 0.022 | 0.053 | no |
| ENG | endoglin | 4.958 | 0.053 | 0.032 | 0.037 | no |
| PPM1B | protein phosphatase, Mg2+/Mn2+ dependent 1B | 4.382 | 0.053 | 0.032 | 0.146 | no |
| WFIKKN2 | WAP, follistatin/kazal, immunoglobulin, kunitz and netrin domain containing 2 | 8.395 | 0.053 | 0.019 | 0.037 | no |
| TNFRSF19 | TNF receptor superfamily member 19 | 7.427 | 0.052 | 0.024 | 0.037 | no |
| EDIL3 | EGF like repeats and discoidin domains 3 | 4.529 | 0.052 | 0.028 | 0.040 | no |
| EDA2R | ectodysplasin A2 receptor | 7.816 | 0.052 | 0.016 | 0.045 | no |
| CDNF | cerebral dopamine neurotrophic factor | 4.242 | 0.052 | 0.033 | 0.041 | no |
| WNT9A | Wnt family member 9A | 5.684 | 0.052 | 0.025 | 0.044 | no |
| RELT | RELT, TNF receptor | 7.776 | 0.052 | 0.025 | 0.038 | no |
| PAMR1 | peptidase domain containing associated with muscle regeneration 1 | 8.935 | 0.052 | 0.020 | 0.035 | no |
| PGLYRP1 | peptidoglycan recognition protein 1 | 10.026 | 0.052 | 0.024 | 0.037 | no |
| CPE | carboxypeptidase E | 8.687 | 0.052 | 0.019 | 0.041 | no |
| CLEC4A | C-type lectin domain family 4 member A | 7.645 | 0.052 | 0.020 | 0.041 | no |
| CD5 | CD5 molecule | 8.073 | 0.052 | 0.018 | 0.038 | no |
| SIGLEC6 | sialic acid binding Ig like lectin 6 | 7.690 | 0.051 | 0.018 | 0.056 | no |
| PDGFC | platelet derived growth factor C | 6.784 | 0.051 | 0.020 | 0.040 | no |
| TGFA | transforming growth factor alpha | 5.728 | 0.051 | 0.026 | 0.039 | no |
| NT5E | 5'-nucleotidase ecto | 13.054 | 0.051 | 0.019 | 0.042 | no |
| LTA | lymphotoxin alpha | 7.821 | 0.051 | 0.019 | 0.055 | no |
| CCL14 | C-C motif chemokine ligand 14 | 7.904 | 0.051 | 0.023 | 0.040 | no |
| SIGLEC1 | sialic acid binding Ig like lectin 1 | 8.574 | 0.051 | 0.017 | 0.041 | no |
| OSMR | oncostatin M receptor | 5.297 | 0.051 | 0.028 | 0.041 | no |
| SIGLEC7 | sialic acid binding Ig like lectin 7 | 6.872 | 0.051 | 0.021 | 0.037 | no |
| THPO | thrombopoietin | 7.526 | 0.051 | 0.027 | 0.035 | no |
| REG1A | regenerating family member 1 alpha | 9.809 | 0.050 | 0.022 | 0.049 | no |
| PROC | protein C, inactivator of coagulation factors Va and VIIIa | 7.807 | 0.050 | 0.026 | 0.034 | yes |
| GAS6 | growth arrest specific 6 | 7.489 | 0.050 | 0.025 | 0.038 | no |
| PTPRF | protein tyrosine phosphatase, receptor type F | 8.139 | 0.050 | 0.024 | 0.035 | no |
| CDH1 | cadherin 1 | 8.012 | 0.050 | 0.025 | 0.041 | no |
| MET | MET proto-oncogene, receptor tyrosine kinase | 5.779 | 0.050 | 0.029 | 0.036 | yes |
| NECTIN4 | nectin cell adhesion molecule 4 | 9.067 | 0.050 | 0.016 | 0.034 | no |
| CNTN1 | contactin 1 | 7.977 | 0.050 | 0.025 | 0.036 | no |
| SDC1 | syndecan 1 | 10.543 | 0.050 | 0.018 | 0.036 | no |
| BTN3A2 | butyrophilin subfamily 3 member A2 | 6.741 | 0.049 | 0.024 | 0.041 | no |
| CD58 | CD58 molecule | 6.716 | 0.049 | 0.027 | 0.032 | no |
| TIE1 | tyrosine kinase with immunoglobulin like and EGF like domains 1 | 5.196 | 0.049 | 0.023 | 0.037 | no |
| DPP4 | dipeptidyl peptidase 4 | 8.831 | 0.049 | 0.018 | 0.034 | yes |
| SEMA3F | semaphorin 3F | 6.438 | 0.049 | 0.024 | 0.037 | no |
| LAYN | layilin | 8.587 | 0.049 | 0.019 | 0.036 | no |
| MDK | midkine | 9.850 | 0.049 | 0.027 | 0.039 | no |
| CDH3 | cadherin 3 | 8.780 | 0.049 | 0.014 | 0.035 | no |
| PLAU | plasminogen activator, urokinase | 7.902 | 0.049 | 0.024 | 0.051 | yes |
| TNFRSF12A | TNF receptor superfamily member 12A | 9.541 | 0.049 | 0.027 | 0.037 | no |
| CCL2 | C-C motif chemokine ligand 2 | 7.028 | 0.049 | 0.027 | 0.046 | yes |
| SPINT1 | serine peptidase inhibitor, Kunitz type 1 | 5.645 | 0.049 | 0.027 | 0.034 | no |
| CD59 | CD59 molecule (CD59 blood group) | 5.590 | 0.049 | 0.027 | 0.040 | no |
| SCARF1 | scavenger receptor class F member 1 | 11.574 | 0.049 | 0.026 | 0.038 | no |
| TNFRSF11A | TNF receptor superfamily member 11a | 9.630 | 0.048 | 0.020 | 0.033 | no |
| SERPINA7 | serpin family A member 7 | 7.856 | 0.048 | 0.023 | 0.036 | no |
| IL18R1 | interleukin 18 receptor 1 | 11.581 | 0.048 | 0.016 | 0.033 | no |
| DCBLD2 | discoidin, CUB and LCCL domain containing 2 | 7.648 | 0.048 | 0.022 | 0.037 | no |
| LGMN | legumain | 8.155 | 0.048 | 0.019 | 0.035 | no |
| SCARB2 | scavenger receptor class B member 2 | 7.066 | 0.048 | 0.019 | 0.034 | no |
| TPP1 | tripeptidyl peptidase 1 | 8.243 | 0.048 | 0.025 | 0.040 | no |
| ATP6AP2 | ATPase H+ transporting accessory protein 2 | 4.426 | 0.048 | 0.029 | 0.038 | no |
| VSIG4 | V-set and immunoglobulin domain containing 4 | 9.478 | 0.047 | 0.017 | 0.038 | no |
| AGER | advanced glycosylation end-product specific receptor | 8.941 | 0.047 | 0.015 | 0.033 | no |
| SPOCK1 | SPARC/osteonectin, cwcv and kazal like domains proteoglycan 1 | 6.657 | 0.047 | 0.020 | 0.037 | no |
| ESAM | endothelial cell adhesion molecule | 7.905 | 0.047 | 0.027 | 0.038 | no |
| CD163 | CD163 molecule | 11.103 | 0.047 | 0.021 | 0.036 | no |
| EPHA2 | EPH receptor A2 | 6.413 | 0.047 | 0.024 | 0.035 | yes |
| WIF1 | WNT inhibitory factor 1 | 9.244 | 0.047 | 0.020 | 0.036 | no |
| SIGLEC10 | sialic acid binding Ig like lectin 10 | 7.352 | 0.047 | 0.016 | 0.037 | no |
| CD1C | CD1c molecule | 9.084 | 0.047 | 0.017 | 0.035 | no |
| CPA2 | carboxypeptidase A2 | 13.261 | 0.047 | 0.016 | 0.037 | no |
| CXCL1 | C-X-C motif chemokine ligand 1 | 13.502 | 0.047 | 0.022 | 0.045 | no |
| CD200 | CD200 molecule | 8.720 | 0.046 | 0.016 | 0.035 | no |
| CXCL13 | C-X-C motif chemokine ligand 13 | 10.682 | 0.046 | 0.019 | 0.035 | no |
| EBI3 | Epstein-Barr virus induced 3 | 9.280 | 0.046 | 0.013 | 0.036 | no |
| CRHBP | corticotropin releasing hormone binding protein | 7.878 | 0.046 | 0.021 | 0.037 | no |
| TFPI2 | tissue factor pathway inhibitor 2 | 11.810 | 0.046 | 0.018 | 0.033 | no |
| IL17D | interleukin 17D | 6.482 | 0.046 | 0.025 | 0.040 | no |
| EPHB6 | EPH receptor B6 | 7.623 | 0.046 | 0.019 | 0.035 | no |
| ACAN | aggrecan | 6.766 | 0.046 | 0.020 | 0.035 | no |
| MRC2 | mannose receptor C type 2 | 5.228 | 0.046 | 0.023 | 0.033 | no |
| MATN2 | matrilin 2 | 9.373 | 0.046 | 0.018 | 0.037 | no |
| ROBO1 | roundabout guidance receptor 1 | 7.073 | 0.046 | 0.025 | 0.031 | no |
| WISP1 | WNT1 inducible signaling pathway protein 1 | 9.623 | 0.046 | 0.020 | 0.037 | no |
| HS6ST1 | heparan sulfate 6-O-sulfotransferase 1 | 7.517 | 0.046 | 0.022 | 0.046 | no |
| GPC1 | glypican 1 | 9.012 | 0.045 | 0.019 | 0.033 | no |
| CCL23 | C-C motif chemokine ligand 23 | 13.046 | 0.045 | 0.019 | 0.033 | no |
| GFRA3 | GDNF family receptor alpha 3 | 7.250 | 0.045 | 0.017 | 0.039 | no |
| CD28 | CD28 molecule | 4.409 | 0.045 | 0.023 | 0.053 | no |
| ADGRE5 | adhesion G protein-coupled receptor E5 | 9.168 | 0.045 | 0.019 | 0.034 | no |
| IGSF3 | immunoglobulin superfamily member 3 | 7.124 | 0.045 | 0.019 | 0.045 | no |
| SMOC2 | SPARC related modular calcium binding 2 | 11.583 | 0.045 | 0.016 | 0.032 | no |
| EGFL7 | EGF like domain multiple 7 | 8.093 | 0.045 | 0.019 | 0.037 | no |
| PRSS27 | serine protease 27 | 12.785 | 0.045 | 0.014 | 0.030 | no |
| IL18BP | interleukin 18 binding protein | 9.415 | 0.045 | 0.021 | 0.032 | no |
| EFNA4 | ephrin A4 | 6.124 | 0.045 | 0.022 | 0.034 | no |
| SELE | selectin E | 15.075 | 0.045 | 0.015 | 0.032 | no |
| CLEC5A | C-type lectin domain containing 5A | 7.775 | 0.044 | 0.019 | 0.032 | no |
| F7 | coagulation factor VII | 8.243 | 0.044 | 0.022 | 0.035 | yes |
| TGM2 | transglutaminase 2 | 12.387 | 0.044 | 0.028 | 0.035 | no |
| CX3CL1 | C-X3-C motif chemokine ligand 1 | 9.199 | 0.044 | 0.018 | 0.036 | no |
| CCL13 | C-C motif chemokine ligand 13 | 16.764 | 0.044 | 0.020 | 0.031 | no |
| CCL11 | C-C motif chemokine ligand 11 | 10.951 | 0.044 | 0.016 | 0.030 | no |
| MSR1 | macrophage scavenger receptor 1 | 9.182 | 0.044 | 0.015 | 0.035 | no |
| PODXL2 | podocalyxin like 2 | 9.877 | 0.044 | 0.013 | 0.036 | no |
| LAMA4 | laminin subunit alpha 4 | 7.556 | 0.044 | 0.025 | 0.035 | no |
| PLAUR | plasminogen activator, urokinase receptor | 8.189 | 0.043 | 0.022 | 0.033 | yes |
| TACSTD2 | tumor associated calcium signal transducer 2 | 8.065 | 0.043 | 0.015 | 0.033 | no |
| TNFRSF1B | TNF receptor superfamily member 1B | 9.033 | 0.043 | 0.021 | 0.035 | no |
| FASLG | Fas ligand | 12.601 | 0.043 | 0.012 | 0.031 | no |
| MERTK | MER proto-oncogene, tyrosine kinase | 10.096 | 0.043 | 0.015 | 0.029 | no |
| NECTIN2 | nectin cell adhesion molecule 2 | 9.507 | 0.043 | 0.020 | 0.030 | no |
| ANPEP | alanyl aminopeptidase, membrane | 8.326 | 0.043 | 0.021 | 0.032 | no |
| ITGA5 | integrin subunit alpha 5 | 7.733 | 0.043 | 0.020 | 0.032 | no |
| LRP1 | LDL receptor related protein 1 | 6.698 | 0.042 | 0.020 | 0.041 | no |
| KLK11 | kallikrein related peptidase 11 | 10.445 | 0.042 | 0.015 | 0.033 | no |
| PDGFRA | platelet derived growth factor receptor alpha | 8.079 | 0.042 | 0.015 | 0.034 | yes |
| CA12 | carbonic anhydrase 12 | 6.975 | 0.042 | 0.018 | 0.049 | yes |
| LPL | lipoprotein lipase | 13.855 | 0.042 | 0.013 | 0.028 | yes |
| FAM3C | family with sequence similarity 3 member C | 9.645 | 0.042 | 0.021 | 0.032 | no |
| COLEC12 | collectin subfamily member 12 | 9.358 | 0.042 | 0.020 | 0.031 | no |
| NOTCH1 | notch 1 | 5.779 | 0.042 | 0.024 | 0.029 | no |
| BCAM | basal cell adhesion molecule (Lutheran blood group) | 7.920 | 0.042 | 0.020 | 0.031 | no |
| ADM | adrenomedullin | 11.320 | 0.042 | 0.018 | 0.031 | no |
| TNFRSF9 | TNF receptor superfamily member 9 | 10.342 | 0.042 | 0.016 | 0.034 | no |
| FAS | Fas cell surface death receptor | 8.728 | 0.042 | 0.023 | 0.034 | no |
| IL1R2 | interleukin 1 receptor type 2 | 9.116 | 0.042 | 0.021 | 0.031 | no |
| ROBO2 | roundabout guidance receptor 2 | 9.550 | 0.041 | 0.016 | 0.032 | no |
| ENTPD6 | ectonucleoside triphosphate diphosphohydrolase 6 (putative) | 6.060 | 0.041 | 0.017 | 0.039 | no |
| B4GAT1 | beta-1,4-glucuronyltransferase 1 | 8.710 | 0.041 | 0.017 | 0.029 | no |
| ADAM8 | ADAM metallopeptidase domain 8 | 7.173 | 0.041 | 0.019 | 0.034 | no |
| TNFRSF1A | TNF receptor superfamily member 1A | 9.651 | 0.041 | 0.021 | 0.029 | yes |
| S100A11 | S100 calcium binding protein A11 | 6.413 | 0.041 | 0.022 | 0.031 | no |
| LYPD3 | LY6/PLAUR domain containing 3 | 8.293 | 0.041 | 0.017 | 0.032 | no |
| CLEC14A | C-type lectin domain containing 14A | 9.161 | 0.041 | 0.021 | 0.029 | no |
| COMP | cartilage oligomeric matrix protein | 10.769 | 0.041 | 0.019 | 0.033 | no |
| KLK8 | kallikrein related peptidase 8 | 10.907 | 0.041 | 0.019 | 0.031 | no |
| CNTN4 | contactin 4 | 8.899 | 0.041 | 0.016 | 0.031 | no |
| LY9 | lymphocyte antigen 9 | 9.233 | 0.041 | 0.015 | 0.030 | no |
| ESM1 | endothelial cell specific molecule 1 | 13.418 | 0.040 | 0.011 | 0.028 | no |
| FOLR1 | folate receptor 1 | 10.786 | 0.040 | 0.016 | 0.029 | no |
| TINAGL1 | tubulointerstitial nephritis antigen like 1 | 7.135 | 0.040 | 0.019 | 0.027 | no |
| LRPAP1 | LDL receptor related protein associated protein 1 | 11.431 | 0.040 | 0.021 | 0.037 | no |
| LIFR | LIF receptor alpha | 7.173 | 0.040 | 0.021 | 0.026 | no |
| FURIN | furin, paired basic amino acid cleaving enzyme | 12.968 | 0.040 | 0.015 | 0.032 | no |
| TFPI | tissue factor pathway inhibitor | 12.379 | 0.039 | 0.018 | 0.025 | yes |
| SEMA7A | semaphorin 7A (John Milton Hagen blood group) | 9.449 | 0.039 | 0.015 | 0.025 | no |
| PEAR1 | platelet endothelial aggregation receptor 1 | 9.712 | 0.039 | 0.023 | 0.029 | no |
| CD99L2 | CD99 molecule like 2 | 8.469 | 0.039 | 0.020 | 0.025 | no |
| METRNL | meteorin like, glial cell differentiation regulator | 7.929 | 0.039 | 0.019 | 0.027 | no |
| VEGFD | vascular endothelial growth factor D | 12.348 | 0.039 | 0.011 | 0.033 | no |
| NPDC1 | neural proliferation, differentiation and control 1 | 9.245 | 0.039 | 0.022 | 0.028 | no |
| CTSL | cathepsin L | 10.646 | 0.039 | 0.016 | 0.028 | no |
| CKAP4 | cytoskeleton associated protein 4 | 7.882 | 0.039 | 0.020 | 0.027 | no |
| ICAM1 | intercellular adhesion molecule 1 | 9.897 | 0.039 | 0.019 | 0.029 | yes |
| FLT3LG | fms related tyrosine kinase 3 ligand | 12.580 | 0.038 | 0.015 | 0.025 | no |
| TGFBI | transforming growth factor beta induced | 11.291 | 0.038 | 0.018 | 0.027 | no |
| CFHR5 | complement factor H related 5 | 11.301 | 0.038 | 0.017 | 0.033 | no |
| FGFBP1 | fibroblast growth factor binding protein 1 | 9.175 | 0.038 | 0.014 | 0.041 | no |
| TNFRSF10B | TNF receptor superfamily member 10b | 8.619 | 0.038 | 0.017 | 0.025 | no |
| SCARF2 | scavenger receptor class F member 2 | 9.748 | 0.038 | 0.013 | 0.028 | no |
| CST3 | cystatin C | 10.157 | 0.038 | 0.021 | 0.029 | no |
| PREB | prolactin regulatory element binding | 5.201 | 0.038 | 0.022 | 0.045 | no |
| NCAN | neurocan | 11.129 | 0.038 | 0.013 | 0.027 | no |
| TGFBR2 | transforming growth factor beta receptor 2 | 10.279 | 0.038 | 0.015 | 0.027 | no |
| RARRES1 | retinoic acid receptor responder 1 | 5.569 | 0.038 | 0.021 | 0.059 | no |
| CD200R1 | CD200 receptor 1 | 7.950 | 0.037 | 0.015 | 0.037 | no |
| CDON | cell adhesion associated, oncogene regulated | 7.818 | 0.037 | 0.023 | 0.029 | no |
| UNC5C | unc-5 netrin receptor C | 8.799 | 0.037 | 0.018 | 0.032 | no |
| C2 | complement C2 | 8.655 | 0.037 | 0.019 | 0.038 | no |
| CD48 | CD48 molecule | 9.742 | 0.037 | 0.014 | 0.028 | no |
| IGF1R | insulin like growth factor 1 receptor | 7.279 | 0.037 | 0.020 | 0.031 | yes |
| THOP1 | thimet oligopeptidase 1 | 8.888 | 0.037 | 0.019 | 0.031 | no |
| CANT1 | calcium activated nucleotidase 1 | 8.606 | 0.036 | 0.018 | 0.023 | no |
| PODXL | podocalyxin like | 6.885 | 0.036 | 0.013 | 0.025 | no |
| SIGLEC9 | sialic acid binding Ig like lectin 9 | 8.577 | 0.036 | 0.011 | 0.031 | no |
| HGF | hepatocyte growth factor | 10.308 | 0.036 | 0.016 | 0.028 | no |
| APOM | apolipoprotein M | 10.622 | 0.036 | 0.016 | 0.024 | no |
| PRSS8 | serine protease 8 | 12.896 | 0.036 | 0.013 | 0.025 | no |
| ANG | angiogenin | 10.852 | 0.036 | 0.019 | 0.039 | no |
| IL10RB | interleukin 10 receptor subunit beta | 8.922 | 0.035 | 0.014 | 0.026 | no |
| MMP7 | matrix metallopeptidase 7 | 13.055 | 0.035 | 0.011 | 0.029 | yes |
| GFRA1 | GDNF family receptor alpha 1 | 10.117 | 0.035 | 0.014 | 0.026 | no |
| RGMB | repulsive guidance molecule BMP co-receptor b | 10.137 | 0.035 | 0.013 | 0.026 | no |
| VEGFA | vascular endothelial growth factor A | 13.167 | 0.034 | 0.016 | 0.025 | yes |
| RGMA | repulsive guidance molecule BMP co-receptor a | 11.412 | 0.034 | 0.012 | 0.025 | no |
| TNFRSF13B | TNF receptor superfamily member 13B | 12.745 | 0.034 | 0.011 | 0.026 | no |
| ERBB4 | erb-b2 receptor tyrosine kinase 4 | 9.017 | 0.034 | 0.012 | 0.025 | yes |
| CD4 | CD4 molecule | 8.634 | 0.034 | 0.018 | 0.026 | yes |
| NRP2 | neuropilin 2 | 8.379 | 0.034 | 0.017 | 0.025 | no |
| DCN | decorin | 8.891 | 0.034 | 0.015 | 0.025 | no |
| EPHB4 | EPH receptor B4 | 8.757 | 0.034 | 0.018 | 0.027 | no |
| CDH6 | cadherin 6 | 7.638 | 0.034 | 0.013 | 0.028 | no |
| IGF2R | insulin like growth factor 2 receptor | 10.808 | 0.033 | 0.016 | 0.031 | no |
| BOC | BOC cell adhesion associated, oncogene regulated | 8.484 | 0.033 | 0.017 | 0.024 | no |
| FST | follistatin | 14.811 | 0.033 | 0.018 | 0.026 | no |
| HMOX1 | heme oxygenase 1 | 15.475 | 0.033 | 0.010 | 0.024 | no |
| AXL | AXL receptor tyrosine kinase | 12.290 | 0.033 | 0.014 | 0.023 | no |
| AMIGO2 | adhesion molecule with Ig like domain 2 | 7.816 | 0.033 | 0.015 | 0.024 | no |
| TIMP1 | TIMP metallopeptidase inhibitor 1 | 8.979 | 0.033 | 0.018 | 0.026 | no |
| F3 | coagulation factor III, tissue factor | 10.556 | 0.032 | 0.012 | 0.027 | yes |
| CD300C | CD300c molecule | 9.046 | 0.032 | 0.010 | 0.028 | no |
| SELL | selectin L | 10.803 | 0.032 | 0.015 | 0.023 | no |
| GALNT2 | polypeptide N-acetylgalactosaminyltransferase 2 | 8.180 | 0.032 | 0.014 | 0.027 | no |
| SERPINA5 | serpin family A member 5 | 11.464 | 0.031 | 0.016 | 0.024 | no |
| PRTG | protogenin | 9.731 | 0.031 | 0.014 | 0.026 | no |
| F11 | coagulation factor XI | 10.777 | 0.031 | 0.014 | 0.024 | yes |
| FOLR3 | folate receptor 3 | 10.136 | 0.031 | 0.011 | 0.142 | no |
| CD27 | CD27 molecule | 12.450 | 0.030 | 0.009 | 0.023 | no |
| ICOSLG | inducible T cell costimulator ligand | 9.590 | 0.030 | 0.015 | 0.024 | no |
| KITLG | KIT ligand | 12.904 | 0.030 | 0.010 | 0.021 | no |
| ITGB1 | integrin subunit beta 1 | 11.248 | 0.029 | 0.018 | 0.021 | yes |
| EZR | ezrin | 8.956 | 0.029 | 0.014 | 0.022 | no |
| PVR | poliovirus receptor | 10.840 | 0.029 | 0.010 | 0.022 | no |
| IFNGR1 | interferon gamma receptor 1 | 8.927 | 0.028 | 0.012 | 0.023 | yes |
| JAM2 | junctional adhesion molecule 2 | 11.792 | 0.028 | 0.012 | 0.020 | no |
| TNFSF10 | TNF superfamily member 10 | 11.840 | 0.028 | 0.013 | 0.021 | no |
| THBD | thrombomodulin | 13.692 | 0.028 | 0.013 | 0.020 | no |
| MARCO | macrophage receptor with collagenous structure | 10.474 | 0.028 | 0.010 | 0.021 | no |
| LGALS9 | galectin 9 | 12.772 | 0.027 | 0.009 | 0.023 | no |
| SORT1 | sortilin 1 | 11.898 | 0.027 | 0.015 | 0.021 | no |
| TNFSF13 | TNF superfamily member 13 | 11.502 | 0.027 | 0.012 | 0.022 | no |
| ACVRL1 | activin A receptor like type 1 | 11.419 | 0.026 | 0.011 | 0.019 | no |
| FLT4 | fms related tyrosine kinase 4 | 11.191 | 0.026 | 0.009 | 0.021 | yes |
| DNER | delta/notch like EGF repeat containing | 12.459 | 0.026 | 0.011 | 0.019 | no |
| ERBB2 | erb-b2 receptor tyrosine kinase 2 | 12.051 | 0.025 | 0.010 | 0.019 | yes |
| DDR1 | discoidin domain receptor tyrosine kinase 1 | 9.771 | 0.025 | 0.011 | 0.018 | no |
| THBS2 | thrombospondin 2 | 10.173 | 0.025 | 0.011 | 0.019 | no |
| DLL1 | delta like canonical Notch ligand 1 | 12.689 | 0.024 | 0.010 | 0.018 | no |
| NTRK3 | neurotrophic receptor tyrosine kinase 3 | 11.541 | 0.024 | 0.010 | 0.018 | no |
| CTSS | cathepsin S | 9.367 | 0.024 | 0.010 | 0.019 | no |
| KDR | kinase insert domain receptor | 10.813 | 0.024 | 0.010 | 0.017 | yes |
| WFDC2 | WAP four-disulfide core domain 2 | 11.654 | 0.023 | 0.011 | 0.018 | no |
| PGF | placental growth factor | 11.797 | 0.023 | 0.012 | 0.017 | yes |
| CPM | carboxypeptidase M | 11.910 | 0.023 | 0.007 | 0.016 | no |
| SELPLG | selectin P ligand | 9.909 | 0.023 | 0.009 | 0.026 | no |
| ERBB3 | erb-b2 receptor tyrosine kinase 3 | 11.506 | 0.023 | 0.011 | 0.018 | no |
| TEK | TEK receptor tyrosine kinase | 11.704 | 0.022 | 0.009 | 0.016 | yes |
| LGALS1 | galectin 1 | 10.769 | 0.022 | 0.011 | 0.017 | no |
| OSCAR | osteoclast associated, immunoglobulin-like receptor | 14.238 | 0.022 | 0.008 | 0.017 | no |
| TNFRSF21 | TNF receptor superfamily member 21 | 12.224 | 0.022 | 0.008 | 0.016 | no |
| SCARA5 | scavenger receptor class A member 5 | 11.197 | 0.022 | 0.009 | 0.016 | no |
| MIA | melanoma inhibitory activity | 13.886 | 0.021 | 0.007 | 0.016 | no |
| SMAD5 | SMAD family member 5 | 7.936 | 0.019 | 0.011 | 0.016 | no |
| CSF1 | colony stimulating factor 1 | 13.094 | 0.019 | 0.011 | 0.015 | no |
| PRELP | proline and arginine rich end leucine rich repeat protein | 10.341 | 0.019 | 0.009 | 0.014 | no |
| GPNMB | glycoprotein nmb | 10.690 | 0.018 | 0.009 | 0.014 | no |
| SPARC | secreted protein acidic and cysteine rich | 9.852 | 0.017 | 0.010 | 0.013 | no |
| PIGR | polymeric immunoglobulin receptor | 10.088 | 0.017 | 0.007 | 0.012 | no |
| AMBP | alpha-1-microglobulin/bikunin precursor | 11.456 | 0.017 | 0.008 | 0.012 | no |
| NRCAM | neuronal cell adhesion molecule | 12.073 | 0.016 | 0.008 | 0.013 | no |
| NBL1 | neuroblastoma 1, DAN family BMP antagonist | 9.233 | 0.015 | 0.008 | 0.036 | no |
| CEACAM1 | carcinoembryonic antigen related cell adhesion molecule 1 | 10.186 | 0.015 | 0.008 | 0.011 | no |
| SPON2 | spondin 2 | 14.088 | 0.014 | 0.008 | 0.009 | no |

**Table S3: A list of 186 proteins with at least a 10% contribution from a certain environmental component**

| Protein | Biomarker group | Fraction (%) | Protein | Biomarker group | Fraction (%) | Protein | Biomarker group | Fraction (%) |
| --- | --- | --- | --- | --- | --- | --- | --- | --- |
| IL1RN | Acute phase | 0.101 | CCL7 | Kidney | 0.144 | RNASE3 | Leukocytes | 0.120 |
| MMP12 | Acute phase | 0.168 | CXCL9 | Kidney | 0.251 | SKAP1 | Leukocytes | 0.212 |
| IL6 | Acute phase | 0.107 | CST5 | Kidney | 0.101 | GH1 | Lipid profile | 0.112 |
| IL10 | Acute phase | 0.145 | IL15RA | Kidney | 0.105 | REN | Lipid profile | 0.109 |
| NCAN | Body composition | 0.170 | IL10RB | Kidney | 0.114 | LPL | Lipid profile | 0.199 |
| RGMA | Body composition | 0.116 | CD5 | Kidney | 0.128 | AGRP | Lipid profile | 0.131 |
| SMPD1 | Body composition | 0.108 | CXCL10 | Kidney | 0.234 | LDLR | Lipid profile | 0.456 |
| BCAN | Body composition | 0.143 | TNFRSF9 | Kidney | 0.202 | TFPI | Lipid profile | 0.159 |
| LAYN | Body composition | 0.101 | CSF1 | Kidney | 0.101 | MPO | Lipid profile | 0.112 |
| GFRA3 | Body composition | 0.119 | TNFRSF1B | Kidney | 0.256 | SCGB3A2 | Lipid profile | 0.190 |
| CPM | Body composition | 0.188 | IL2RA | Kidney | 0.134 | CYR61 | Lipid profile | 0.132 |
| IL1RN | Body composition | 0.228 | IL18BP | Kidney | 0.194 | CLEC4A | Lipid profile | 0.142 |
| GDF2 | Body composition | 0.113 | MMP3 | Kidney | 0.273 | HSD11B1 | Lipid profile | 0.102 |
| LEP | Body composition | 0.752 | TNFRSF1A | Kidney | 0.255 | MASP1 | Lipid profile | 0.106 |
| FABP4 | Body composition | 0.345 | TGFBR2 | Kidney | 0.145 | NOS3 | Lipid profile | 0.113 |
| SELE | Body composition | 0.151 | TGFA | Kidney | 0.140 | CES2 | Lipid profile | 0.239 |
| IGFBP1 | Body composition | 0.295 | CD27 | Kidney | 0.152 | NPPC | Lipid profile | 0.102 |
| PLAT | Body composition | 0.107 | DLL1 | Kidney | 0.145 | TMPRSS15 | Lipid profile | 0.126 |
| PON3 | Body composition | 0.218 | TNFRSF19 | Kidney | 0.143 | CTSO | Lipid profile | 0.133 |
| IGFBP2 | Body composition | 0.233 | CD160 | Kidney | 0.205 | GHRL | Lipid profile | 0.142 |
| FURIN | Body composition | 0.260 | TNFRSF4 | Kidney | 0.176 | CDHR5 | Lipid profile | 0.142 |
| SEZ6L | Body composition | 0.127 | LILRB4 | Kidney | 0.206 | ENTPD5 | Lipid profile | 0.119 |
| HGF | Body composition | 0.140 | CLEC4D | Kidney | 0.185 | CLUL1 | Lipid profile | 0.215 |
| WIF1 | Body composition | 0.159 | SH2D1A | Kidney | 0.153 | REG4 | Lipid profile | 0.113 |
| SH2D1A | Body composition | 0.102 | NCR1 | Kidney | 0.134 | APOM | Lipid profile | 0.289 |
| ITGB6 | Body composition | 0.119 | CLEC6A | Kidney | 0.173 | PROC | Lipid profile | 0.102 |
| DPP6 | Body composition | 0.145 | CD83 | Kidney | 0.101 | PLA2G7 | Lipid profile | 0.107 |
| PTN | Body composition | 0.204 | BTN3A2 | Kidney | 0.154 | GCG | Lipid profile | 0.107 |
| CLMP | Body composition | 0.316 | NPPC | Kidney | 0.157 | MFGE8 | Lipid profile | 0.131 |
| APLP1 | Body composition | 0.126 | SERPINB8 | Kidney | 0.116 | SPINK1 | Lipid profile | 0.176 |
| ADGRG2 | Body composition | 0.207 | SEMA3F | Kidney | 0.151 | CD300LG | Lipid profile | 0.333 |
| LILRA5 | Body composition | 0.105 | FAM3C | Kidney | 0.104 | CGA | Lipid profile | 0.253 |
| SSC4D | Body composition | 0.267 | NECTIN2 | Kidney | 0.128 | CA2 | Lipid profile | 0.113 |
| CES1 | Body composition | 0.154 | NPDC1 | Kidney | 0.122 | XG | Lipid profile | 0.116 |
| MOG | Body composition | 0.217 | CST3 | Kidney | 0.381 | CD209 | Lipid profile | 0.101 |
| WFIKKN2 | Body composition | 0.173 | IGFBP6 | Kidney | 0.307 | MME | Liver | 0.225 |
| SPON2 | Erythrocytes | 0.114 | LCN2 | Kidney | 0.104 | KYNU | Liver | 0.279 |
| PAPPA | Erythrocytes | 0.119 | ZBTB17 | Kidney | 0.105 | ACE2 | Liver | 0.208 |
| TGM2 | Erythrocytes | 0.113 | FAM19A5 | Kidney | 0.140 | HAO1 | Liver | 0.211 |
| MB | Erythrocytes | 0.178 | PILRA | Kidney | 0.103 | CDCP1 | Liver | 0.101 |
| CA1 | Erythrocytes | 0.270 | SIRPB1 | Kidney | 0.187 | CCL19 | Liver | 0.100 |
| PROK1 | Erythrocytes | 0.308 | VSIG4 | Kidney | 0.184 | ANPEP | Liver | 0.155 |
| SCGB3A1 | Erythrocytes | 0.132 | FCER2 | Kidney | 0.140 | NT5E | Liver | 0.344 |
| NPPB | Heart | 0.636 | RELT | Kidney | 0.244 | NFATC3 | Liver | 0.223 |
| TNNI3 | Heart | 0.148 | PGF | Kidney | 0.166 | MVK | Liver | 0.140 |
| NT-proBNP | Heart | 0.654 | CSF3 | Leukocytes | 0.117 | THOP1 | Liver | 0.206 |
| CD300C | Kidney | 0.148 | OLR1 | Leukocytes | 0.257 | ALDH1A1 | Liver | 0.261 |
| EZR | Kidney | 0.122 | CEACAM8 | Leukocytes | 0.239 | FBP1 | Liver | 0.135 |
| EFNA4 | Kidney | 0.250 | IL17A | Leukocytes | 0.105 | VAMP5 | Liver | 0.212 |
| SCARB2 | Kidney | 0.178 | OSM | Leukocytes | 0.436 | SULT2A1 | Liver | 0.154 |
| CD38 | Kidney | 0.397 | MMP9 | Leukocytes | 0.369 | VWC2 | Platelets | 0.103 |
| RGMB | Kidney | 0.176 | AZU1 | Leukocytes | 0.178 | SORT1 | Platelets | 0.139 |
| GFRA1 | Kidney | 0.117 | PRTN3 | Leukocytes | 0.116 | SERPINE1 | Platelets | 0.129 |
| SCARA5 | Kidney | 0.143 | PGLYRP1 | Leukocytes | 0.161 | PDGFA | Platelets | 0.102 |
| GZMA | Kidney | 0.116 | TCL1A | Leukocytes | 0.123 | INPPL1 | Platelets | 0.102 |
| ACVRL1 | Kidney | 0.165 | ANXA1 | Leukocytes | 0.109 | ERBIN | Platelets | 0.115 |
| JAM2 | Kidney | 0.171 | IL6 | Leukocytes | 0.270 | MCFD2 | Platelets | 0.101 |
| EDA2R | Kidney | 0.150 | EGLN1 | Leukocytes | 0.149 | FLI1 | Platelets | 0.103 |
| ADM | Kidney | 0.114 | CLEC4D | Leukocytes | 0.103 | APP | Platelets | 0.137 |
| TNFRSF11A | Kidney | 0.198 | TREM1 | Leukocytes | 0.192 | CD99L2 | Platelets | 0.102 |
| TNFRSF10B | Kidney | 0.162 | SIT1 | Leukocytes | 0.180 | CD69 | Platelets | 0.115 |
| LGALS9 | Kidney | 0.301 | RASSF2 | Leukocytes | 0.107 | CTRC | Urate | 0.114 |
| THBD | Kidney | 0.107 | NCF2 | Leukocytes | 0.269 | GALNT2 | Urate | 0.134 |
| IL16 | Kidney | 0.175 | NADK | Leukocytes | 0.154 |  |  |  |
